# pH Controlled Activation and Stabilization of Enzymes Using Responsive Polymer-Bioconjugates

**DOI:** 10.1021/acs.biomac.5c00212

**Published:** 2025-06-06

**Authors:** Monica S. Rahman, Bhagya Chandrarathne, Katie Bender, Jasmine Hinkle, Richard C. Page, Dominik Konkolewicz

**Affiliations:** Department of Chemistry and Biochemistry, 6403 Miami University, 651 E High Street, Oxford, Ohio 45056, United States

## Abstract

Stimuli-responsive bioconjugates are developed to control enzymatic activity and stability using a pH-responsive polymer based on *N*,*N*-dimethyl aminopropylacrylamide. Prior work has shown that conjugating *N*-isopropylacrylamide (NIPAm) polymer to theCandida antarctica lipase B (CalB) enzyme has an inhibitory effect. Due to the similar hydrophobicity to the substrate of CalB, NIPAm polymer may block the enzyme’s catalytic site, thereby inhibiting the enzyme’s activity. This study shows that conjugating pDMAPA DP40-*b*-NIPAm DP10 to CalB has a pH-responsive effect on the catalytic property of CalB, with inverse stability responses. At low pH, where the polymer is extended, high activity is observed, while at low pH, the inhibitory block binds or occludes the active site and increases stability. A similar approach used a known inhibitor, *N*-acryloyl-d-glucosamine (AGA), for lysozyme. Conjugating pDMAPA DP40-*b*-AGA DP10 elicited a similar pH-controlled stability–activity response in lysozyme. Therefore, we propose that the polymer’s pH-dependent interaction with protein can effectively regulate the protein between a highly active state and high stability behavior.

## Graphical abstract

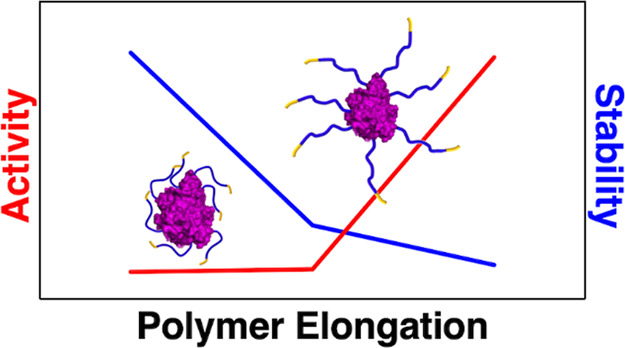

## Introduction

Protein–polymer conjugation has been studied extensively for its diverse applications in medicine, biotechnology, and nanotechnology. Conjugates resulting from the covalent attachment of synthetic polymers to a protein can provide improved stability, solubility, and biocompatibility.^1,2^ Moreover, synthetic polymer conjugation can introduce new properties into the biomolecules, such as self-assembly and complex phase behavior.^3–6^ The first protein–polymer conjugate was reported in 1977 by Abuchowski et al., who conjugated poly­(ethylene glycol) (PEG) to protein drugs, showing prolonged circulatory times in the body with enhanced drug efficacy.^7–9^ Since then, investigations on protein–polymer conjugation have been carried out for more than 40 years to produce different functional and stimuli-responsive “smart conjugates”.^10–14^

There are significant efforts to synthesize complex protein–polymer conjugates.^12,15–19^

Reversible deactivation radical polymerization (RDRP) methods are well suited to attach functional polymers on the protein surface and impact the activity and stability of proteins.^10,20–23^ The most common RDRP approaches for bioconjugation include reversible addition–fragmentation chain transfer (RAFT) polymerization^24^ and atom-transfer radical polymerization.^25^ Synthesis of polymers using these methods produces well-defined polymers with complex architecture.^26–28^

For conjugation, “grafting to” is a powerful synthetic approach for attaching presynthesized polymers to the biomolecule. Essentially all PEGylated protein therapeutics have been produced through “grafting to” technology.^29–31^ The functional end of the synthesized polymer is then attached to the protein surface using well-established reactions with either naturally occurring amino acid residues or incorporated reactive handles.^32^ Despite its simplicity, “grafting to” can face challenges of low grafting density due to steric obstacles and subsequent purification after conjugation.^18,33^ On the other hand, the “grafting from” strategy attaches a small molecule initiator or chain transfer agent to the protein surface with subsequent in situ polymer growth in the presence of a monomer and an appropriate solvent.^11,18,34,35^ “Grafting-from” facilitates purification and increases grafting density.^18,36^ However, “grafting-from” can have limited conditions suitable for polymerization and protein stability and presents challenges associated with characterization.^37^

Stimuli-responsive or “smart” protein–polymer conjugates have received significant attention over the last few decades,^38^ with their early reports being evident in the late 1970s and early 1980s.^39–42^ They have identified the application of carboxylated polymers in phase separation using either low pH or calcium ions.^39–42^ In early 1980, Hoffman and co-workers used temperature-responsive polymers such as poly­(*N*-isopropylacrylamide) (poly­(NIPAm)) to synthesize conjugates with a monoclonal antibody.^43–46^ The introduction of such a temperature-responsive polymer demonstrated thermally induced phase separation in immunoassay.^44,46,47^ Subsequently, many researchers have used “smart” polymers to tune protein behavior for diverse applications ranging from bioseparation, biosensing, drug delivery, tuning enzyme activity, immunoassays, and affinity precipitation.^13,48–53^

With their physical or chemical changes, responsive polymers can either self-assemble or undergo phase transition, conformational rearrangement, or morphology changes. These polymers can be sensitive to stimuli ranging from temperature, pH, chemical reagents, solvent, ionic strength, light, electric field, and a magnetic field.^5,54,55^ Among all stimuli-responsive polymers, pH-responsive polymers have gained significant recognition in the last two decades in both academic and commercial fields, considering both synthetic methodologies and wide-span applications.^56^ Most commonly, “pH-responsive polymers” consist of either acidic or basic residues that change their charge in response to the pH of the solution.^57–59^ Acidic pH-responsive polymers are typically weak poly acids (for example, poly­(methacrylic acid)­(PMAAc)) that are neutral at low pH and release protons at high pH, yielding negatively charged polymers. On the other hand, basic pH-responsive polymers are weak poly bases (for example, poly­(*N*,*N*-dimethyl aminopropylacrylamide) (poly­(DMAPA))), which accept protons at low pH, forming a positively charged polymer, while being neutrally charged at higher pH.^56^

One of the most important reasons to use stimuli-responsive polymers or conjugates is their ability to control drug release, which holds significant potential for the development of efficient drug delivery systems.^6^ Several synthetic polymers can be used as pH-responsive polymers, such as poly­(aspartic acid), which is used for colon-targeted drug delivery, and poly­(glutamic acid), which targets breast cancer cells.^60^

Due to their targeted drug release capabilities, these systems can reduce off-target drug effects and decrease dosage frequency. Vanparijs et al. developed a self-assembled bioconjugate system using [(2,2-dimethyl-1,3-dioxolane)­methyl]­acrylamide and bovine serum albumin protein. These nanoparticles are responsive to both temperature and pH.^61^

Additionally, Rai et al. developed a pH-responsive bioconjugate system to enhance the stability of urease, using a derivative of urease and poly­(acrylamide) and poly­(2-acrylamido-2-methylpropanesulfonic acid) polymers.^62^

In our previous study,^63^ we observed that the conjugation of Candida antarctica lipase B (CalB) with DMAPA enhances the activity of the enzyme, while conjugation with NiPAm almost completely diminished the enzymatic activity. Conjugation with *N*,*N*-dimethyl acrylamide (DMAm) was also shown to enhance the activity of CalB significantly.^63^ Similarly, when lysozyme conjugates were studied, the highest activity was found when cationic polymers were attached and acryloyl-d-glucosamine (AGA) acted as a potent inhibitor. The NIPAm and AGA polymers might bind to the catalytic sites of CalB and lysozyme, respectively, thereby limiting the access of the substrate and causing inhibition of the enzyme.

This contribution develops stimuli-responsive block polymers with a pH-responsive monomer unit in the first block and an inhibitor for the protein of interest in the second block. Attaching these pH-responsive polymers to inhibitors can regulate the catalytic behavior of proteins. When the pH-responsive poly­(DMAPA) polymer is ionized, the chain is extended, removing the inhibitor from the active site and thus reenabling protein activity. In contrast, when the pH-responsive polymer is uncharged, the inhibitor can noncovalently bind at the active site, reducing activity by occluding the active site but creating a more stable conjugate (Scheme 1D), since the inhibitor noncovalently binds different parts of the protein together.^64^ Since DMAPA polymers are pH-responsive, with a p*K*_a_ near 8.3,^65^ the polymers are primarily cationic at pH < 7.5 and primarily uncharged at pH > 9. Hence, block polymers of DMAPA with inhibitory blocks of either NIPAm or AGA in the second block were prepared to modulate the activity and stability of CalB and lysozyme with pH. Activity is expected to be maximal at low pH when the polymer containing the inhibitory block is in an extended conformation, thereby removing the inhibitor from the active site, allowing enzymatic turnover.

**Scheme 1:**
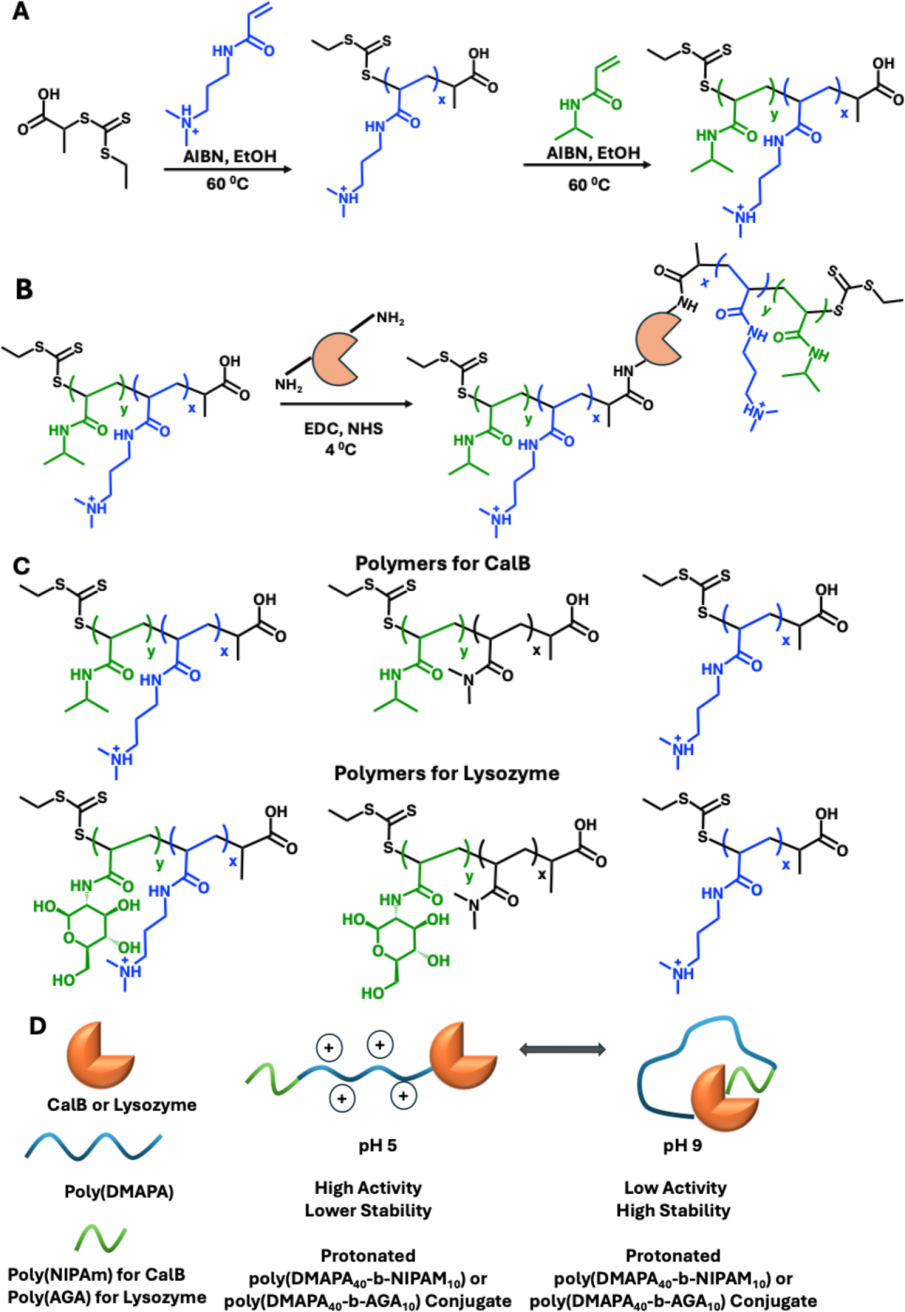
(A) RAFT Polymerization of Poly­(DMAPA_*x*_-*b*-NIPAm_*y*_); (B) “Grafting to” Approach via EDC-NHS Coupling of Poly­(DMAPA_*x*_-*b*-NIPAm_*y*_) to Lipase Protein; (C) Polymer Scaffolds Used in This Study; (D) Higher Activity of Poly­(DMAPA_*x*_-*b*-NIPAm_*y*_) or Poly­(DMAPA_*x*_-*b*-AGA_*y*_) Conjugate at pH 5 Due to Chain Elongation and Lower Activity with Higher Stability at pH 9 Due to Interactions with the Catalytic Site of Either CalB or Lysozyme

The three-dimensional folded structure of many proteins typically involves disparate parts of the primary sequence placed into close proximity and typically only held together by weak noncovalent forces.^66^ For instance, the active site of many enzymes is composed of two or more structural elements held in close proximity only through noncovalent interactions between them. Therefore, binding of an inhibitor to the active site through intermolecular forces increases the number of noncovalent bonds that must be broken to unfold the protein; thereby, noncovalent inhibition can increase stability.^67^ Since at high pH, the NIPAm or AGA polymers can noncovalently bind to the active site of CalB and lysozyme, respectively, this inhibitory block binding can bind disparate structural units and stabilize the system at high pH.

## Experimental Methodology

### Synthesis of PAETC

Polymer synthesis was carried out using the RAFT polymerization approach using the chain transfer agent 2-(((ethylthio)-carbonothioyl)­thio) propionic acid (PAETC). To produce PAETC, an aqueous potassium hydroxide solution (14.6 g, 0.26 mol) was prepared and added dropwise to a stirring mixture of ethanethiol in acetone kept in an ice bath. After stirring for 30 min, carbon disulfide was added to the mixture dropwise for 10 min. After removal from the ice bath, 2-bromo-propionic acid (23.0 mL, 0.26 mol) was added dropwise to the reaction mixture. After that, the reaction mixture was stirred overnight at room temperature. Subsequently, acetone was removed entirely from the mixture by rotary evaporation. The product obtained after evaporation was redissolved in a saturated sodium carbonate solution. After washing with excess diethyl ether, the aqueous layer was acidified and extracted with about 50 mL of diethyl ether three times. Subsequently, the ether layer containing the product was washed with excess ether and water. Finally, the solvent was removed from the product using a reduced pressure. PAETC was obtained as a yellow product.

^1^H NMR spectra for the obtained PAETC were as follows (400 MHz, CDCl_3_, δ ppm): 4.80 (1H, q, *J* = 7.4 Hz, CH_3_CH­(S)–COOH), 3.32 (2H, q, *J* = 7.4 Hz, CH_3_CH_2_S), 1.56 (3H, d, *J* = 7.4 Hz, CH_3_CH­(S)–COOH), 3.32 (2H, q, *J* = 7.4 Hz, CH_3_CH_2_S), 1.56 (3H, d, *J* = 7.4 Hz, CH_3_CH­(S)­COOH), 1.30 (3H, t, *J* = 7.4 Hz, CH_3_CH_2_S).

### Synthesis of Polymer

Each polymer was synthesized by using a RAFT polymerization approach. Synthesis of pDMAPA DP40-*b*-NIPAm DP10 is shown as an example in Scheme 1. PAETC (0.10 g, 0.48 mmol), AIBN (0.01 g, 0.05 mmol), and DMAPA (3.0 g, 19.03. mmol) were dissolved in 5 mL of ethanol in a 50 mL round-bottom flask. The flask was sealed with a rubber cork stopper and deoxygenated with nitrogen sparging for 20 min. Subsequently, the reaction mixture was heated in an oil bath at 65 °C for 21–22 h with continuous stirring. ^1^H NMR spectra were used to calculate the monomer conversion. A 95% or more was considered a satisfactory conversion. To form the second block with DP10 NIPAm polymer, AIBN (0.01 g, 0.05 mmol), NIPAm (0.54 g, 4.76 mmol), and 1 mL ethanol was added to the reaction mixture. After deoxygenation by nitrogen sparging for 20 min, the reaction mixture was heated again in the oil bath at 65 °C for 16 h. With the satisfactory conversion of monomer (<95% or more), the synthesized block polymer was precipitated dropwise in a 120 mL of hexane/ether solvent mixture (80:20). The precipitated polymer was dried in a vacuum for 72 h. The remaining polymers were synthesized using the same protocol.

### Synthesis of *N*-Acryloyl-d-Glucosamine

To synthesize AGA, we adopted the procedure mentioned by Matsuda and Sugawara.^68^ For the synthesis, d-glucosamine hydrochloride (8.60 g, 39.88 mmol) and NaNO_2_ (0.15 g, 2.13 mmol) were added to a 20 mL 2 M aqueous solution of Na_2_CO_3_ in a 250 mL conical flask. The reaction mixture was cooled to 0 °C or below in a salt/ice bath and subsequently kept on a magnetic stirrer. To this mixture was added acryloyl chloride (4.02 g, 44.42 mmol) dropwise under stirring. The reaction mixture was kept under the same conditions for 3 h, ensuring the temperature was below 5 °C throughout the entire time. After that, the reaction mixture was stirred at room temperature for 48 h. Then, the mixture was added dropwise to 200 mL of absolute ethanol and kept in the refrigerator overnight to allow for recrystallization. The precipitated salts were filtered the following morning, and the filtered solution obtained was concentrated under a vacuum. Finally, two consecutive recrystallizations of the resultant product were carried out: first in methanol and then by a methanol and ethyl acetate mixture (1 to 2 ratio). ^1^H NMR spectra of the resultant product were in agreement with the literature.

### Characterization of Polymers

The synthesized polymers were characterized by using ^1^H NMR spectroscopy and gel permeation chromatography (GPC). Initially, monomer conversion was checked with Bruker Advance 400 or 500 MHz NMR spectrometer. The molecular weight and dispersity of the polymer were then assessed using GPC. Sample preparation for GPC was carried out by dissolving 3–6 mg of each polymer in an approximate 1.5 mL volume of DMF with a drop of toluene. Following filtration through a 0.2 μm polytetrafluoroethylene filter, the sample solution was injected into an Agilent 1260 GPC system. The system consisted of a degasser, an isocratic pump, an autosampler, a refractive index detector, 2 x Polargel-M, and guard columns. *N*,*N*-Dimethylformamide (DMF) with 0.1 wt % LiBr was used as an eluent. The flow rate of the eluent was regulated at 1 mL/min at 50 °C. Poly­(methacrylate) standards were used to calibrate the system in the 617,000 to 1010 range.

### Conjugation of Polymers to CalB and Lysozyme

The reactive carboxyl end of polymers was attached to the amine group of CalB (Sigma-Aldrich) and lysozyme (hen egg white lysozyme, GoldBio) using an EDC-NHS coupling reaction via the “grafting to” approach. As an example, conjugation of poly­(*N*,*N*-dimethylaminopropylacrylamide) DP40-*b*-(NIPAm) DP10 (pDMAPA DP40-*b*-NIPAm DP10) to CalB is given in Scheme 1B. For the conjugation, pDMAPA DP40-*b*-NIPAm DP10 (0.17 g, 21.74 μmol), EDC (6.01 mg, 31.35 μmol), and NHS (0.25 mg, 2.18 μmol) were mixed in a 1× phosphate buffer saline (1× PBS) (pH = 7.5) in a glass vial. Once mixed properly, CalB (27 μL of 76 mg/mL stock solution) was added to the solution. The final volume of the solution was adjusted to 1.2 mL with a final CalB concentration of 1.7 mg/mL with 1× PBS (pH = 7.5). The reaction mixture was stirred overnight at 4 °C.

Conjugation of poly­(*N*,*N*-dimethylaminopropylacrylamide) DP40-*b*-(*N*-acryloyl-d-glucosamine) (pDMAPA-*b*-AGA DP10) to lysozyme was carried out as follows. In a glass vial, pDMAPA-*b*-AGA DP10 (0.17 g, 25.18 μmol), EDC (4.83 mg, 25.18 μmol), and NHS (0.58 mg, 5 μmol) were mixed in 1× PBS (pH = 7.5) solution. Accordingly, lysozyme (1 mg, 0.07 μmol) was added to the solution to achieve a final concentration of 1 mg/mL. The reaction mixture was allowed to stir overnight at 4 °C.

To purify the conjugates, each of the samples was diluted to 5 mL volume with Milli-Q water in a 5 mL 10,000 MWCO PES concentration tube. The diluted sample was centrifuged in a 4 °C rotor at 4000 rpm with 5 min intervals. The conjugate solution was repeatedly purified by using the same approach until a clear conjugate solution with a stable UV–vis spectrum was obtained. Accordingly, protein–polymer conjugation was confirmed using sodium dodecyl sulfate-polyacrylamide gel electrophoresis (SDS-PAGE). For the CalB^63^ and lysozyme^22^ conjugates, the number of conjugated polymers was estimated to be in the order of 3 per protein, based on analysis from literature protocols that follow the same methodology.

### CalB Activity Assay

To assess the activity of CalB and its conjugates, we considered the lipolysis ability of the enzyme using the substrate *p*-nitrophenyl palmitate (*p*-NPP). Degradation of *p*-NPP yields *p*-nitrophenol (*p*-NP), the absorbance of which was taken at 405 nm as a measure of the lipolytic activity. Initially, *p*-NPP (6.0 mg, 15.9 μmol) was dissolved in 1.0 mL of dimethyl sulfoxide to obtain a substrate stock solution. For the assay, 10 μL of the enzyme was incubated with the respective buffer solution (pH 5 = 0.07 M C_2_H_3_NaO_2_, 0.03 M CH_3_COOH; pH 7 = 0.137 M NaCl, 0.01 M Na_2_HPO_4_, 0.003 M KCl, 0.002 M KH_2_PO_4_; pH 9 = 0.091 M NaHCO_3_, 0.009 M Na_2_CO_3_) in a PCR tube. After incubation for 30 min at room temperature, 50 μL of *p*-NPP stock solution was added to the mixture. The final enzyme concentration was maintained at 5 μM for both CalB and its conjugates. After that, each sample was incubated at 37 °C for 30 min in a Life Technologies Proflex 3 × 32-Well PCR system. Subsequently, each sample was aliquoted in 50 μL volume on a 384-well plate with a further dilution with 50 μL of volume of PBS. Finally, the absorbance was taken at 405 nm in a BioTek Synergy H1 microplate reader to measure lipolytic activity.

The control assay (Figure S20) was performed by simply mixing CalB and each polymer (final concentration: 5 μM) in the respective buffer solution under the same conditions as the activity assay. Absorbance at 405 nm was recorded using a BioTek Synergy H1 microplate reader.

To determine the functional thermal stability, CalB and its conjugates were incubated in their respective buffer solution (pH 5, pH 7, or pH 9) for three different time intervals (0, 3, and 6 h) at 50 and 60 °C temperature. Accordingly, each incubated sample was subjected to a colorimetric assay, as discussed above.

### Lysozyme Activity Assay

Lysozyme activity was determined using the large substrate lyophilized Micrococcus lysodeikticus (Sigma-Aldrich). Lysozyme-catalyzed lysis of the M. lysodeikticus cell wall can be monitored from the gradual decrease in optical density at 450 nm as a function of time. A solution of the substrate was prepared in respective buffer solution (pH 5 = 0.07 M C_2_H_3_NaO_2_, 0.03 M CH_3_COOH; pH 7 = 0.137 M NaCl, 0.01 M Na_2_HPO_4_, 0.003 M KCl, 0.002 M KH_2_PO_4_; pH 9 = 0.091 M NaHCO_3_, 0.009 M Na_2_CO_3_) to achieve a final optical density around 1.0. The substrate solution was aliquoted in a 50 μL volume in a 384-well plate. Approximately 5 μL of either the enzyme or conjugate was added to the substrate solution. A BioTek Synergy H1 microplate reader was used to record the optical density at 450 nm every 10 s over 10 min for the native lysozyme and 5 min for the lysozyme conjugates. Finally, the optical density was plotted against time to obtain the rate of reaction.

### Thermal Stability Using Differential Scanning Fluorimetry

The thermal stability of native lysozyme and its conjugates was analyzed using differential scanning fluorometry. Each sample was diluted in respective pH buffer (pH 5 = 0.07 M C_2_H_3_NaO_2_, 0.03 M CH_3_COOH; pH 7 = 0.137 M NaCl, 0.01 M Na_2_HPO_4_, 0.003 M KCl, 0.002 M KH_2_PO_4_; pH 9 = 0.091 M NaHCO_3_, 0.009 M Na_2_CO_3_) to attain a final protein concentration of 10 μM. The DSF study was carried out using a CFX96 RT-PCR (Bio-Rad) instrument. Fluorescence was recorded at 570 nm, while the temperature was increased in 0.5 °C increments from 25 to 95 °C with a 5 s hold for equilibration at each temperature step. The data obtained were fitted to a Boltzmann equation using Prism (GraphPad) to determine the melting temperatures of the native lysozyme and its conjugates.

### Estimates of p*K*_a_ and Surface Area and Attachment Sites

According to the surface availability and p*K*_a_ values of the lysine amino acids in CalB, there is a possibility to Lys 13 (p*K*_a_ 7.79), Lys 32 (p*K*_a_ 10.29), and Lys 98 (p*K*_a_ 10.43) to get conjugated.

p*K*_a_ and surface availability of each lysine are analyzed using the following sites: p*K*_a_-Propka (https://www.ddl.unimi.it/vegaol/propka_run.php):^69^ Surface area-GETAREA (https://curie.utmb.edu/getarea.html).^70^

As per prior work, Lysines 33, 97, and the N terminus are modified in Lysozyme.^22^

## Results and Discussion

Initially, a series of block polymers of DMAPA and NIPAm were synthesized as the stimuli-responsive polymers to be conjugated to CalB. Since DMAm is not pH-responsive, a series of block polymers of DMAm and NIPAm were made as control conjugates. For each polymer, the initial target was a degree of polymerization of 20 or 40 units in the first block and 5 or 10 units in the second protein-inhibitory block. All the polymers were synthesized using RAFT.^71^ An illustration of the synthesis of poly­(DMAPA_40_-*b*-NIPAm_10_) polymer is given in Scheme 1A. Polymers were characterized by ^1^H NMR spectroscopy (Figure S1) and size exclusion chromatography (Table S2).

The synthesized polymers were conjugated to CalB by using an in situ 1-ethyl-3-(3-(dimethylamino)­propyl) carbodiimide (EDC)-*N*-hydroxysuccinimide (NHS) coupling reaction. CalB has 18 lysine residues and an N-terminal amine group. For all conjugations, a ratio of 1 to 20 for the amine groups to the polymer’s carboxylic acids was used. As an example, the conjugation of poly­(DMAPA_40_-*b*-NIPAm_10_) to CalB is illustrated in Scheme 1B. SDS-PAGE was used to confirm the conjugation (Figure S2). Successful conjugation was confirmed by a shift of the conjugate’s molecular weight to a higher apparent molecular weight. Subsequently, a centrifuged-based filtration approach was used to purify the conjugates.^72^

The lipolytic activity of CalB and its conjugates was determined in a hydrolysis assay using the substrate *p*-NPP. Hydrolysis of *p*-NPP releases *p*-NP, which absorbs at 405 nm. The p*K*_a_ of poly­(DMAPA) was measured to be 8 (Figure S3), consistent with literature reports of a p*K*_a_ of 8.3.^65^ For the assay, three different pH systems, pH = 5, 7, and 9, were used. The activity of each of the conjugates was normalized to that of wild-type CalB in the respective pH system.

The activities of CalB and its conjugates are illustrated in Figure 1 as a percentage relative activity of CalB. Figure S20 indicates that the free polymers do not alter the enzymatic activity substantially. CalB has optimum activity at pH 7 and the lowest activity at pH 5 (Figure S4). As predicted, the CalB-pDMAm-based conjugates also showed a similar trend as the native enzyme (activity: pH 7 > pH 9 > pH 5). The highest activity for these conjugates was observed at pH 7 and 9, with around 116–128% activity for poly­(DMAm_20_-*b*-NIPAm_10_) conjugate and 97–122% activity for poly­(DMAm_20_-NIPAm_5_) conjugate as compared to CalB at the respective pH. These conjugates exhibited the lowest activity at pH 5, with around 42–44% activity for both conjugates relative to CalB at pH 5.

**Figure 1:**
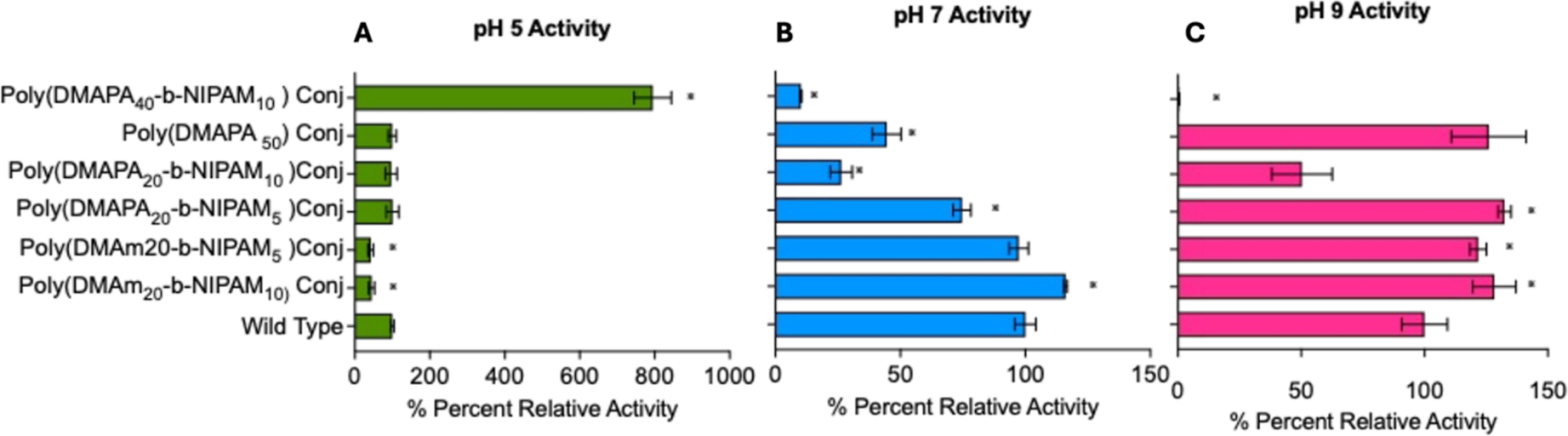
Percent relative activity of wild-type of lipase and its conjugates with poly­(DMAm_20_-*b*-NIPAm_10_), poly­(DMAm_20_-NIPAm_5_), poly­(DMAPA_20_-*b*-NIPAm_5_), poly­(DMAPA_20_-*b*-NIPAM _10_), poly­(DMAPA_40_-*b*-NIPAm_10_), and poly­(DMAPA_50_) at different pH systems. (A) Percent relative activity at pH 5 (green); (B) percent relative activity at pH 7 (blue); and (C) percent relative activity at pH 9 (pink). Activity was normalized with respect to wild-type to lipase at related pH systems. % Percent relative activity = [activity of sample at particular pH systems/activity of wild-type of lipase at that pH] × 100. Each data represents an average of triplicate ± standard deviation. Statical analysis by *t*-test: Paired two samples for mean, compared to WT data at each pH, * indicates *P*-value < 0.05.

However, conjugates with poly­(DMAPA) showed distinct behavior due to the pH-responsive polymer. Poly­(DMAPA_20_-*b*-NIPAm_10_) and poly­(DMAPA_20_-*b*-NIPAm_5_) showed almost the same activity as CalB at pH 5. At higher pH values, poly­(DMAPA_20_-*b*-NIPAm_5_) showed consistently higher activity than poly­(DMAPA_20_-*b*-NIPAm_10_), presumably due to the longer pNIPAm block inhibiting CalB more effectively. In contrast, the poly­(DMAPA_40_-*b*-NIPAm_10_) conjugate gave the desired optimum activity at pH 5 (about 800% of CalB activity at that pH) and diminished activity at pH 9 (0–1% CalB activity). As hypothesized earlier, at lower pH (pH 5), the polymer is charged; hence, it was elongated from the protein surface, keeping away the second block of NIPAm inhibitor. At higher pH (pH 9), the polymer was neutrally charged, which allowed them to collapse on the protein surface, thereby allowing it to noncovalently bind to the catalytic site by the NIPAm inhibitor. Reduced hydrophobic interactions between CalB and NiPAm are also supported by the work of Ao et al.^73^ who found that after covering some of the NiPAm chain with cyclodextrin, the enzymatic activity of CalB increased.

As a control, we also prepared CalB-poly­(DMAPA_50_) conjugate, which showed a similar trend as poly­(DMAPA_20_-*b*-NIPAm_10_) and poly­(DMAPA_20_-*b*-NIPAm_5_) (activity: pH 9 > pH 7 > pH 5). The exceptional activity of the poly­(DMAPA_40_-*b*-NIPAm_10_) at pH = 5 could be in part due to the hydrophobic poly­(NIPAm) block recruiting the hydrophobic *p*-NPP substrate near CalB,^74^ without the polymer obstructing the active site. Comparing the shorter poly­(DMAPA_20_-*b*-NIPAm_10_) and poly­(DMAPA_20_-*b*-NIPAm_5_) polymers to the longer poly­(DMAPA_40_-*b*-NIPAm_10_) suggests that the longer polymer may be more effective at pH 5 due to the longer NIPAm block being more effective at recruiting the substrate and the longer DMAPA40 block in the extended state ensuring the NiPAm block is far from the active site.

With these observations in hand, we used a functional thermal stability assay. If the NIPAm polymer binds the catalytic site of CalB at pH 9, the conjugate could also exhibit enhanced stability at pH 9 compared to that at pH 5. In the stability assay, samples were incubated at two different temperatures, 50 and 60 °C in their respective buffer systems, and their residual activity was evaluated at distinct time points. The stability assay indicates that the native CalB enzyme exhibited optimum stability at pH 7 rather than at pH 5 and 9 in a 6 h incubation period (Figure 2). The CalB conjugates, such as poly­(DMAPA_20_-*b*-NIPAm_10_) conjugate, poly­(DMAPA_20_-*b*-NIPAm_5_) conjugate, poly­(DMAm_20_-*b*-NIPAm_10_) conjugate, and poly­(DMAm_20_-*b*-NIPAm_5_) conjugate exhibited optimum functional thermal stability at pH 5 and lowest stability at pH 9 after 6 h incubation (Figure S5). In these cases, the polymer is likely too short to fully place the inhibitor in the active site at low pH. However, the presence of the ionizable poly­(DMAPA) leads to electrostatic and steric repulsion, which can limit aggregation, enhancing stability. However, CalB-poly­(DMAPA_40_-*b*-NIPAm_10_) conjugate showed considerable functional thermal stability at pH 9 compared to pH 5 and 7, even after 6 h of the incubation period. Essentially no loss of enzymatic activity was observed between 3 and 6 h. This is consistent with a distinct stabilization mechanism where the inhibitory NIPAm block binds the active site, leading to enhanced stability when the poly­(DMAPA) polymer is uncharged and allows the polymer to attach to the active site.

**Figure 2:**
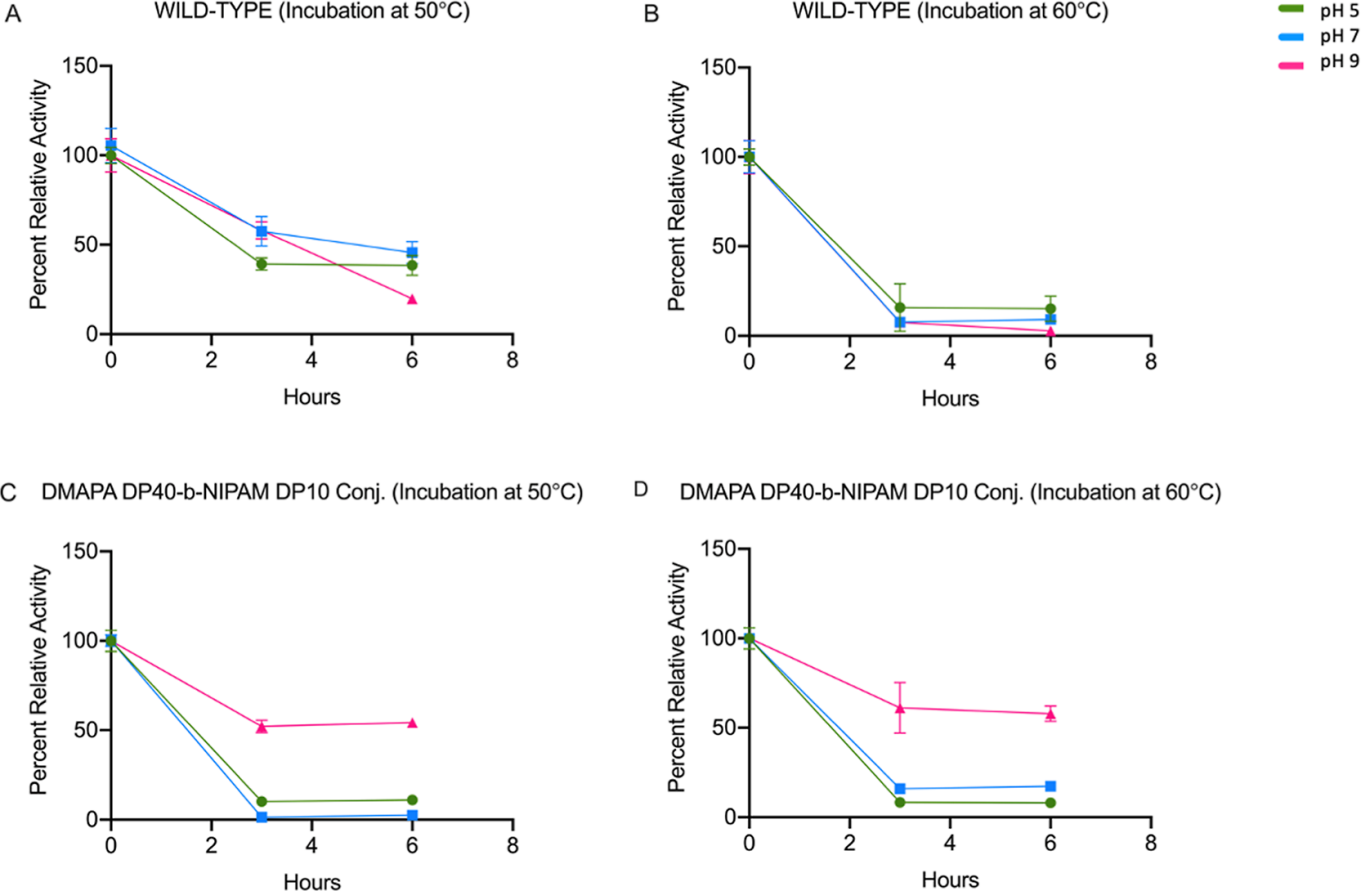
Functional thermal stability of wild-type lipase and its conjugate with poly­(DMAPA_40_-*b*-NIPAm_10_). Each sample was incubated at 50 and 60 °C for two different periods, 3 h and 6 h. The activity of each sample was normalized with respect to their initial activity at zero hours. Activities in pH 5, 7, and 9 are presented in green, blue, and pink, respectively. % Percent relative activity = [absorbance of sample at particular hour/absorbance of sample at zero hour] × 100. Each data represents an average of triplicate ± standard deviation.

Additionally, this approach was also tested on a different model protein, lysozyme, since the pH-responsive behavior of the polymer should be protein-independent. AGA has been shown to be a lysozyme inhibitor.^22^ It is important to note that polymer conjugation to lysozyme can reduce enzymatic activity against the micrococcus substrate due to steric interference.^22^ A diblock copolymer was prepared with DMAPA in the first block and AGA in the second block (poly­(DMAPA_40_-*b*-AGA_10_)). Poly­(DMAm_40_-*b*-AGA_10_) and poly­(DMAPA_50_) polymers were also synthesized as controls. Native lysozyme showed optimum activity at pH 5–6 and lowest activity at pH 9.^75,76^ A similar trend was also observed for the controls, poly­(DMAm _40_-*b*-AGA_10_) and poly­(DMAPA_50_) conjugates. On the other hand, lysozyme-poly­(DMAPA_40_-*b*-AGA_10_) conjugate showed a considerable reduction of activity at pH 9 as compared to native as well as other controls, as seen in Figure 3A.

**Figure 3:**
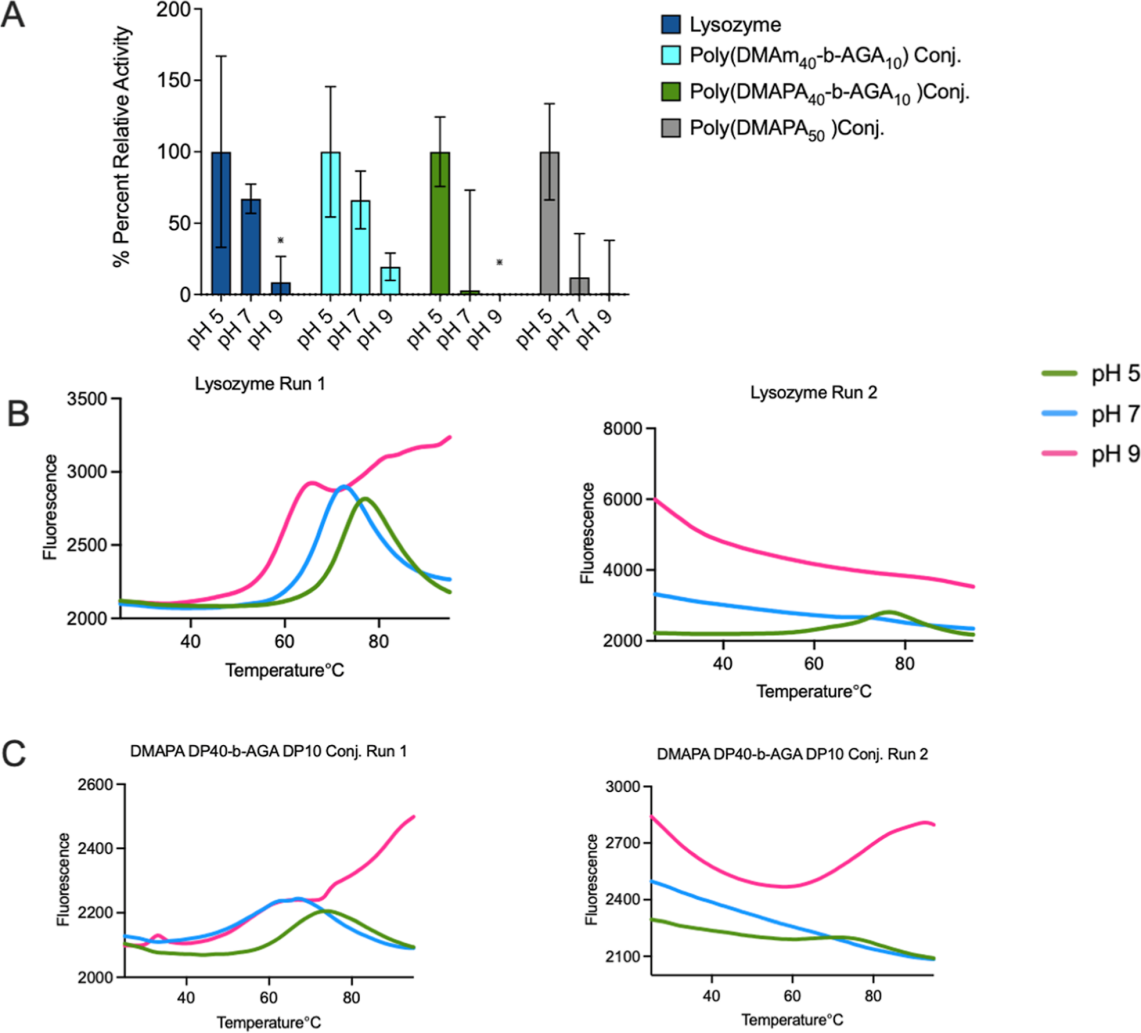
(A) Percent activity reduction of lysozyme and its conjugates with poly­(DMAm_40_-*b*-AGA_10_), poly­(DMAPA_40_-*b*-AGA_10_), and poly­(DMAPA_50_). Each activity was normalized to percent activity reduction with respect to their optimum pH activity. % Percent relative activity = [activity of sample at particular pH/activity of sample at its optimum pH] × 100. Each data represents an average of triplicate ± standard deviation. (B) Differential scanning fluorometry data for wild-type lysozyme protein in the consecutive first and second runs. (C) Differential scanning fluorometry data for poly­(DMAPA_40_-*b*-AGA_10_) conjugate with lysozyme in consecutive first and second runs. In (B,C), fluorescence at pH 5, pH 7, and pH 9 are represented in green, blue, and pink.

The thermal stability of these conjugates was assessed using differential scanning fluorimetry (DSF), as shown in Figure 3B,C. DSF showed that in two consecutive runs, native lysozyme had the highest stability at pH 5 with irreversible aggregation at the other pH values; the lysozyme-poly­(DMAPA_40_-*b*-AGA_10_) conjugate showed better stability than native at pH 9 on the first run and an unfolding peak on the second heating cycle at pH 9, a feature, which is essentially absent or very weak for the native at all pH values studied or the lysozyme-poly­(DMAPA_40_-*b*-AGA_10_) conjugate at lower pH values. This indicates that their lysozyme-poly­(DMAPA_40_-*b*-AGA_10_) conjugate has substantially higher stability at pH 9 compared with pH 5.

## Conclusions

Conjugating a pH-responsive 40-unit long poly­(DMAPA) polymer coupled to a 10-unit long inhibitory block to either CalB or lysozyme can efficiently regulate the catalytic and stability properties of the enzyme. 10-unit long NIPAm and AGA blocks can act as inhibitors for CalB and lysozyme, respectively. At pH ∼ 5, the poly­(DMAPA) block is highly charged, leading the polymer to be extended in solution, pulling the inhibitory NIPAm or AGA block away from the enzyme, facilitating enzymatic activity. Since poly­(DMAPA) is primarily uncharged at pH 9, the data suggests the NIPAm or AGA block binds the enzyme’s catalytic site and reduces the activity while also leading to a concomitant increase in stability at pH = 9 for the inhibitor-containing conjugates. This highlights a new direction for protein–polymer bioconjugate design for tailoring both activity and stability. Creating enzyme bioconjugates that are more stable through reversible inhibition could allow enzymes that are stored under conditions with the inhibitor bound for long-term storage, with enzymatic activity restored after changing the polymer conformation to remove the inhibitor and restore activity. The ability to stabilize a protein with temporary or controlled inhibition may provide value to the pharmaceutical industry or to nonhealth-related industrial applications that utilize enzymes. Previously, the typical approach to stabilizing proteins, such as those used as pharmaceuticals, involved conducting large-scale searches to identify the conditions that both stabilize the protein and maintain activity.^77^ In contrast, temporary pH-controlled binding to the active site, as demonstrated in this work, could provide substantial gains in stability and allow for the restoration of activity upon reverting to a pH that removes inhibition. In this scenario, activity is inhibited temporarily during storage, yet the mode of inhibition allows for substantial gains in stability, a parameter that is arguably more important during storage than activity. This approach could be useful as the activity of the protein during storage is immaterial, as long as activity can be restored at the desired time by exposure to the necessary stimulus (e.g., altering the pH). Thus, pH-controlled activation and stabilization of proteins may provide great value to applications in which the need for enhanced stability and restoration of activity can or will be temporally distanced.

## Supplementary Material



## Notes

Reagent details; supplemental polymer characterization data, titration, nuclear magnetic resonance data, GPC data, and molecular weight data; and supplemental bioconjugate characterization data, polyacrylamide gel electrophoresis data, activity, and stability data (PDF)
