# Supplementary material for: pH Controlled Activation and Stabilization of Enzymes Using Responsive Polymer-Bioconjugates
Source: Biomacromolecules. 2025 Jun 6;26(7):4209–18. doi: 10.1021/acs.biomac.5c00212 (PMC12266049; doi:10.1021/acs.biomac.5c00212)
Supplement: Supplementary file 1 [file bm5c00212_si_001.pdf]

Supporting Information: pH Controlled Activation and Stabilization of Enzymes using Responsive Polymer-Bioconjugates

Monica S. Rahman,[a*] Bhagya Chandrarathne,[a] Katie Bender,[a] Jasmine Hinkle,[a] Richard C. Page,[a*] Dominik Konkolewicz[a*]

[a] Department of Chemistry and Biochemistry, Miami University, 651 E High St, Oxford, OH 45056, USA

Correspondence:

rahmanms@MiamiOH.edu (Monica Sharfin Rahman)

d.konkolewicz@MiamiOH.edu (Dominik Konkolewicz)

pagerc@MiamiOH.edu (Richard C. Page)

List of Reagents Used

Table S1. Reagent used in the study

Reagents	Vendors
N,N-dimethyl acrylamide (DMAm)	TCI
N-[3-(dimethylamino)propyl)acrylamide (DMAPA)	TCI
N-isopropyl acrylamide (NIPAm)	Acros Organics
Ethanethiol	Acros Organics
Azobis(isobutyronitrile) (AIBN)	Sigma Aldrich
2-bromopropionic acid	Thermo Fisher Scientific
Potassium hydroxide	Thermo Fisher Scientific
Carbon disulfide	Fisher Chemical
1-Ethyl-3-(3-(dimethylamino)propyl) carbodiimide (EDC)	Carbosynth

N-hydroxysuccinimide (NHS)	Thermo Scientific
D-glucosamine hydrochloride	Alfa Aesar
Sodium nitrite	Alfa Aesar
Acryloyl chloride	Sigma-Aldrich
Sodium carbonate anhydrous	Tedia
Lipase from Candida antarctica lipase B	Strem Chemicals
p-nitrophenyl palmitate (p-NPP)	Sigma-Aldrich
Lysozyme	MP Biomedicals
Micrococcus lysodeikticus	Sigma-Aldrich

Experimental Data

A

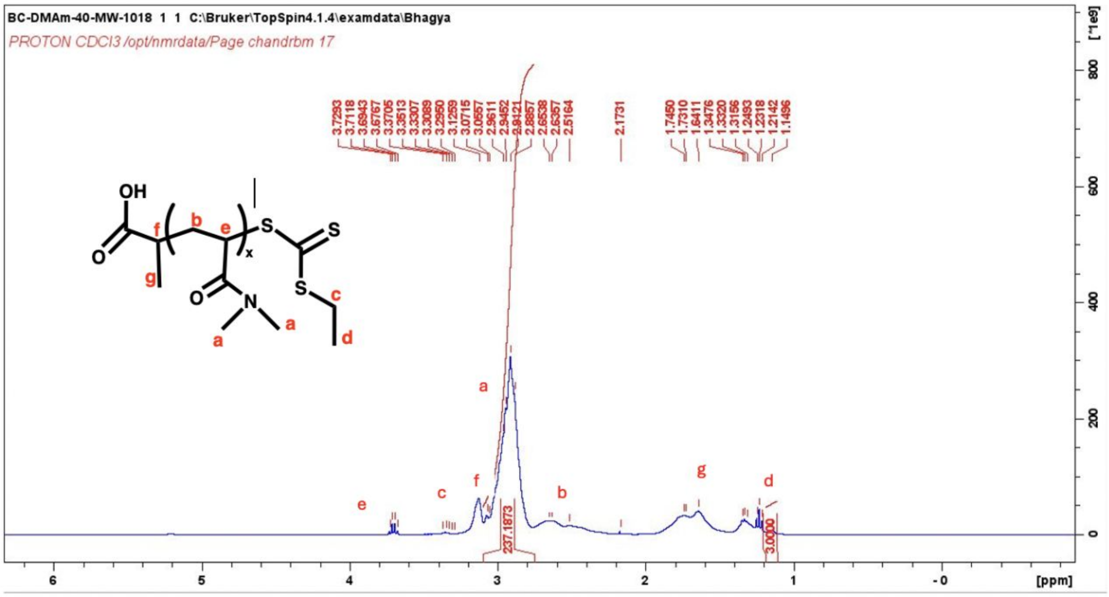

B

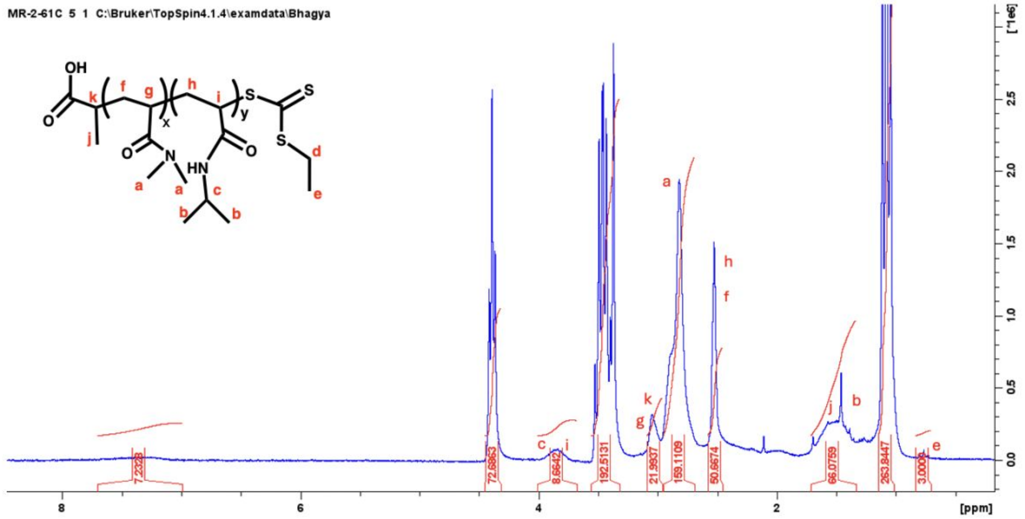

C

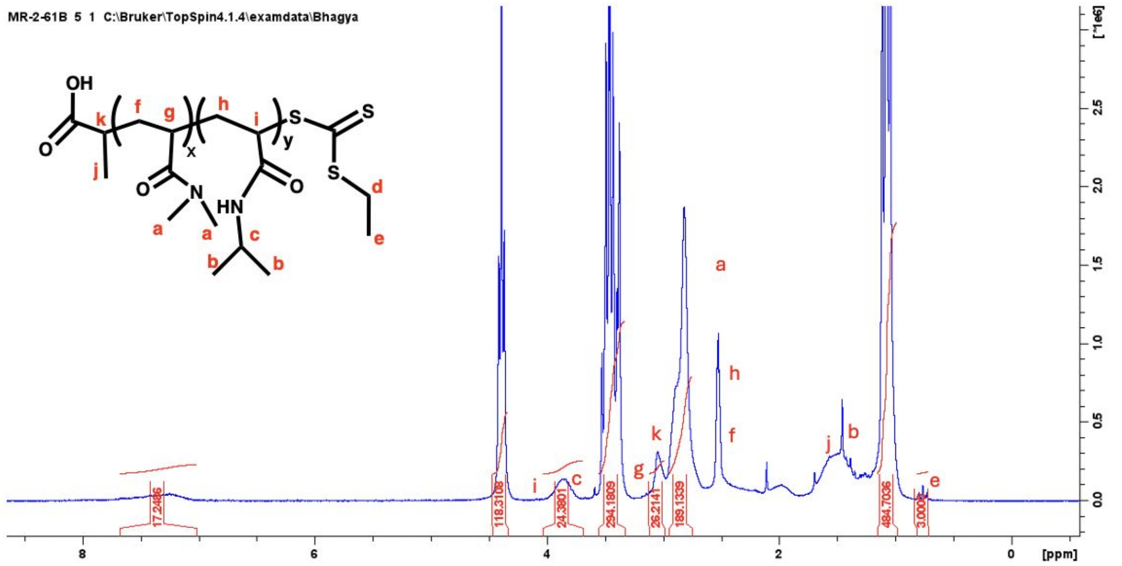
MR-2-61B 5 1 C:\Bruker\TopSpin4.1.4\examdata\Bhagya
[ppm]

D

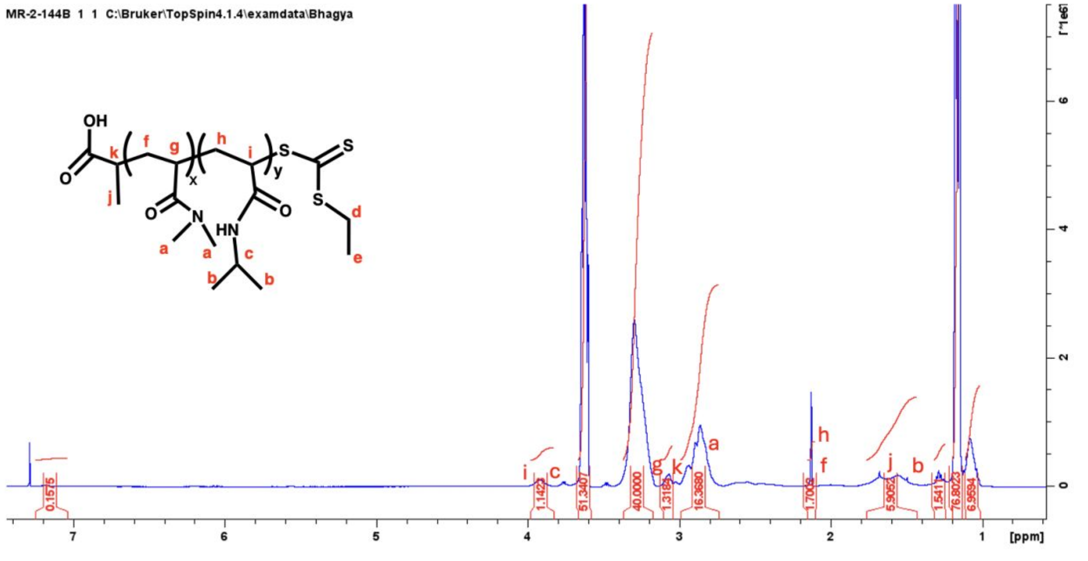
MR-2-144B 1 1 C:\Bruker\TopSpin4.1.4\examdata\Bhagya
[ppm]

E

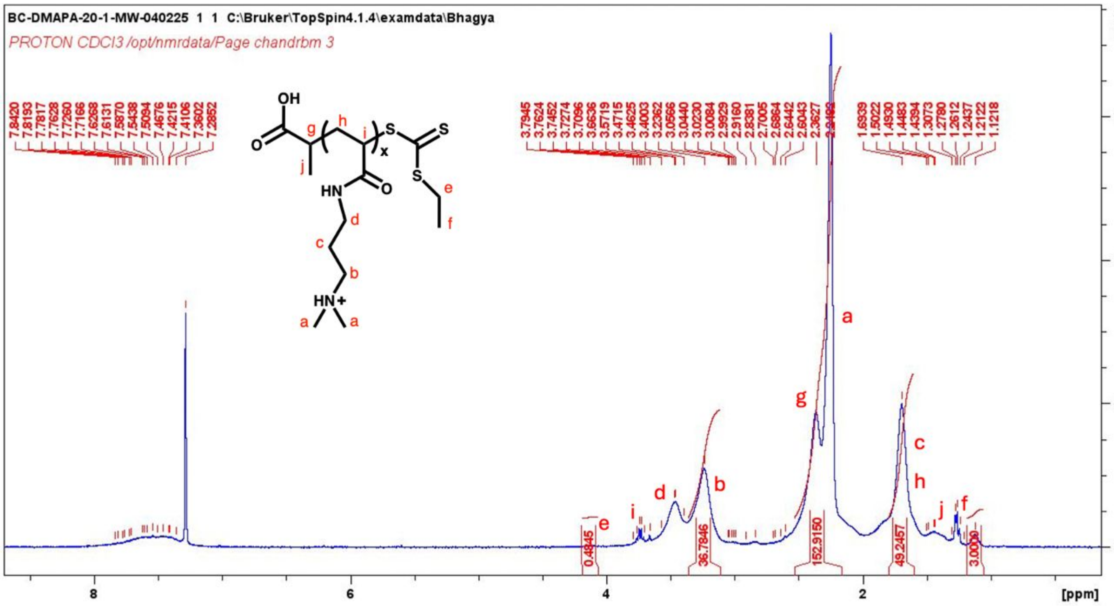
BC-DMAPA-20-1-MW-040225 1 1 C:\Bruker\TopSpin4.1.4\examdata\Bhagya
PROTON CDCl3 /opt/nmrdata/Page chandrbm 3
[*1e9]
OH
S
S
S
HN
O
O
HN+
x
a
b
c
d
e
f
g
h
i
j
0.4845
36.7846
152.9150
49.2457
3.0000
8
6
4
2
[ppm]
0
20
40
60

F

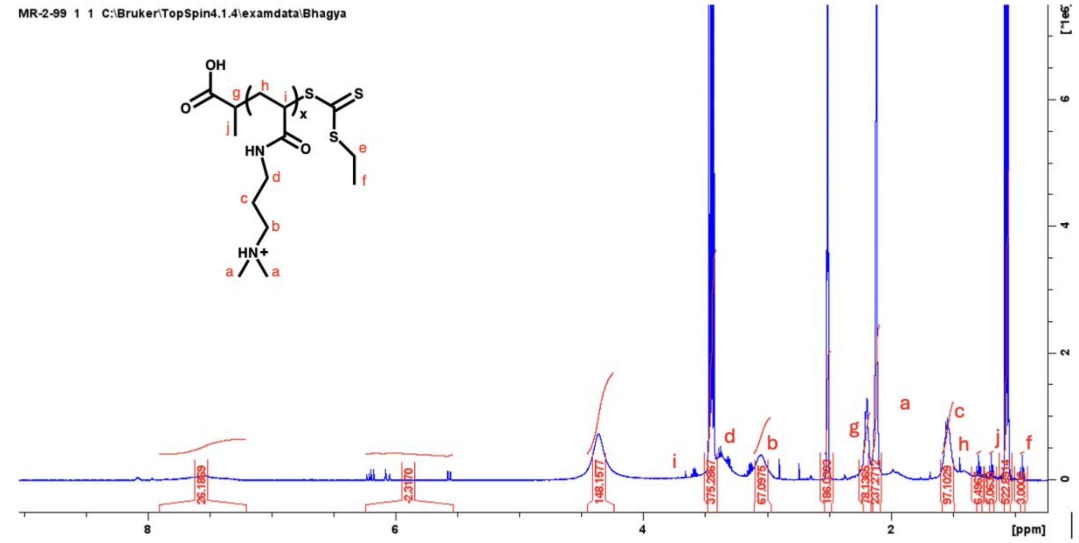
MR-2-99 1 1 C:\Bruker\TopSpin4.1.4\examdata\Bhagya
[*1e6]
OH
S
S
S
HN
O
O
HN+
x
a
b
c
d
e
f
g
h
i
j
26.1869
-2.3170
148.1577
375.2867
67.0975
186.0593
78.1365
237.2712
97.1029
3.0000
8
6
4
2
[ppm]
0
2
4
6

G

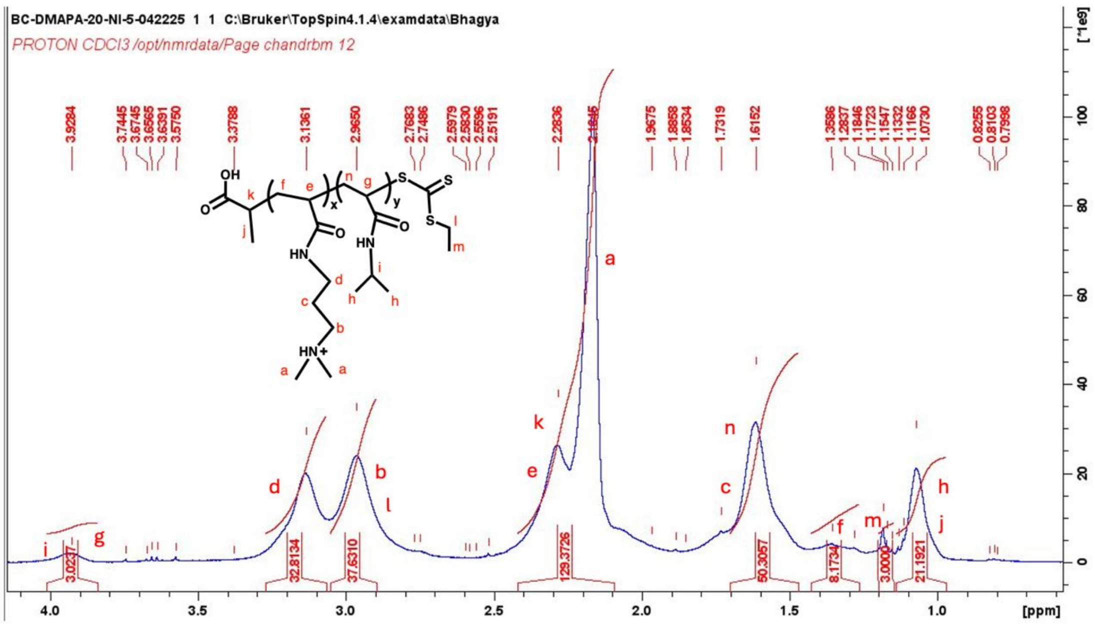

BC-DMAPA-20-NI-5-042225 1 1 C:\Bruker\TopSpin4.1.4\examdata\Bhagya
PROTON CDCl3 /opt/nmrdata/Page chandrbm 12
[*1e9]
[ppm]

H

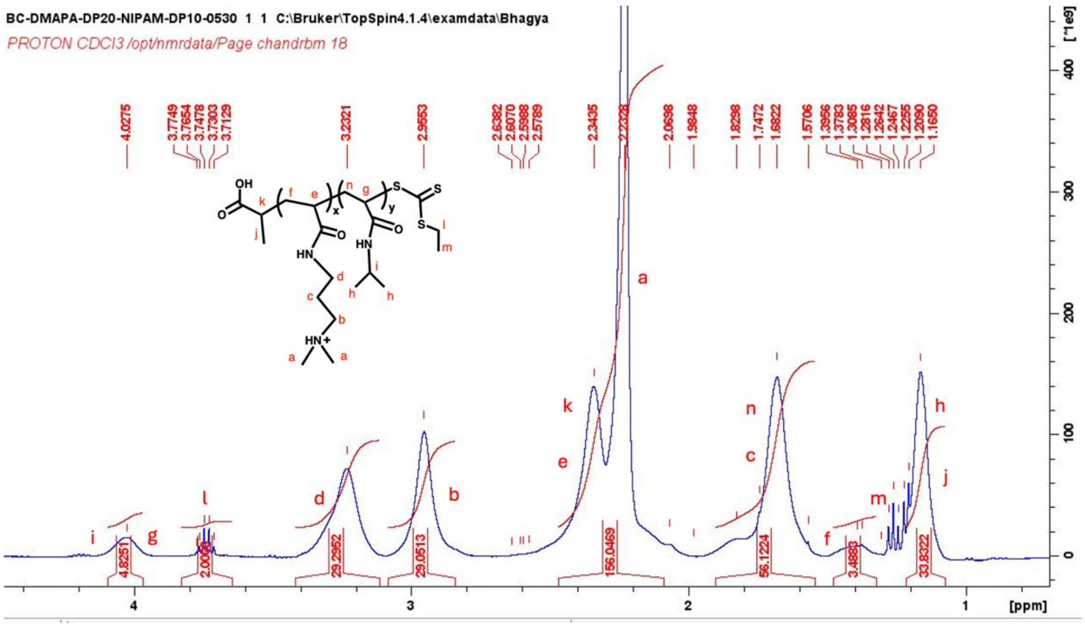

BC-DMAPA-DP20-NIPAM-DP10-0530 1 1 C:\Bruker\TopSpin4.1.4\examdata\Bhagya
PROTON CDCl3 /opt/nmrdata/Page chandrbm 18
[*1e9]
[ppm]

I

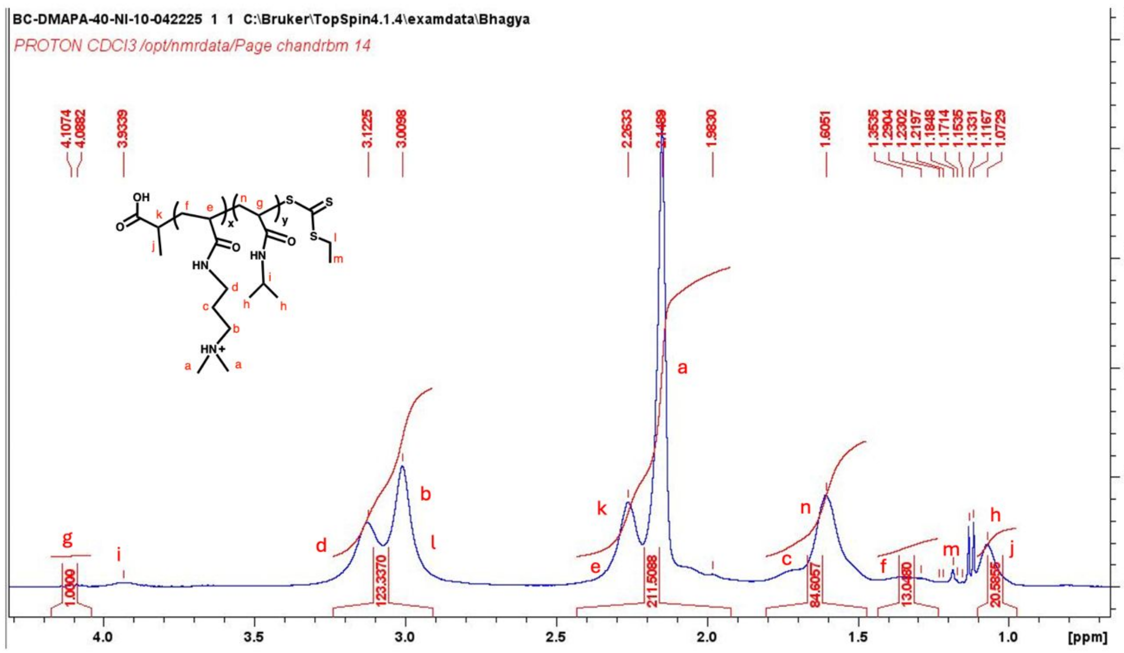
BC-DMAPA-40-NI-10-042225 1 1 C:\Bruker\TopSpin4.1.4\examdata\Bhagya
PROTON CDCl3 /opt/nmrdata/Page chandrbm 14
4.1074
4.0882
3.9339
3.1225
3.0098
2.2633
2.1489
1.9830
1.6051
1.3535
1.2904
1.2302
1.2197
1.1848
1.1714
1.1535
1.1331
1.1167
1.0729
1.0000
123.3370
211.5088
84.6057
13.0480
20.5855
[ppm]
[*1e9]

J

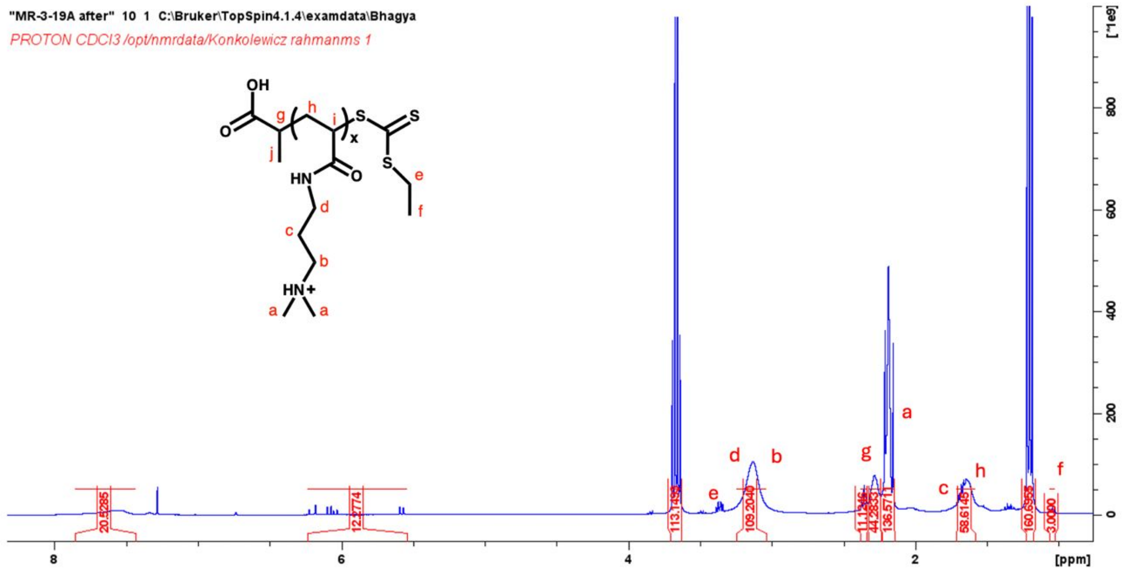
"MR-3-19A after" 10 1 C:\Bruker\TopSpin4.1.4\examdata\Bhagya
PROTON CDCl3 /opt/nmrdata/Konkolewicz rahmanms 1
20.5285
12.3774
113.1492
109.2040
136.571
58.6145
160.6953
3.0000
[ppm]
[*1e9]

K

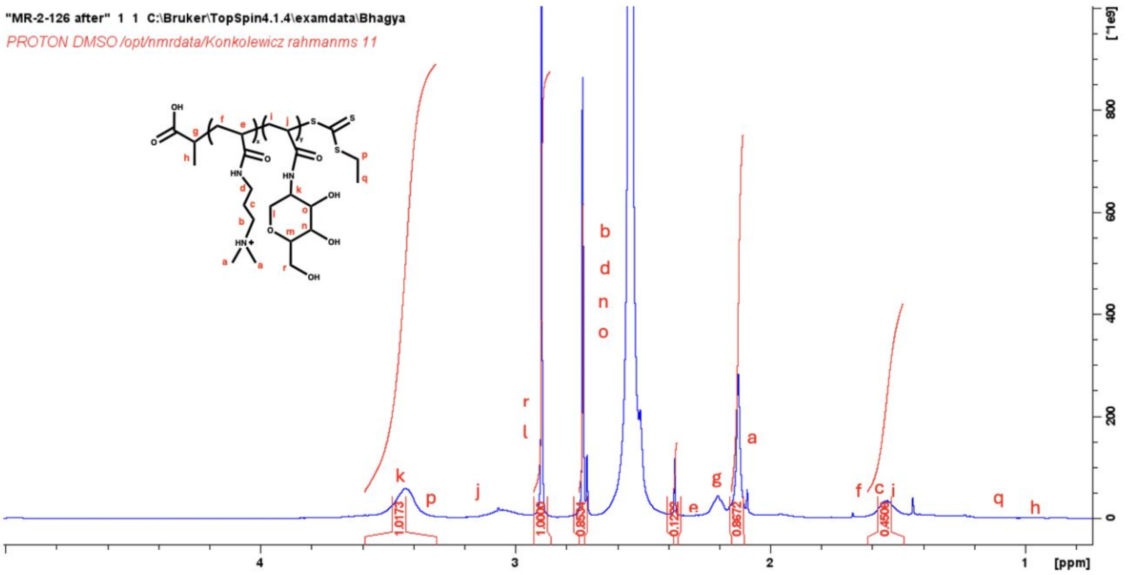

L

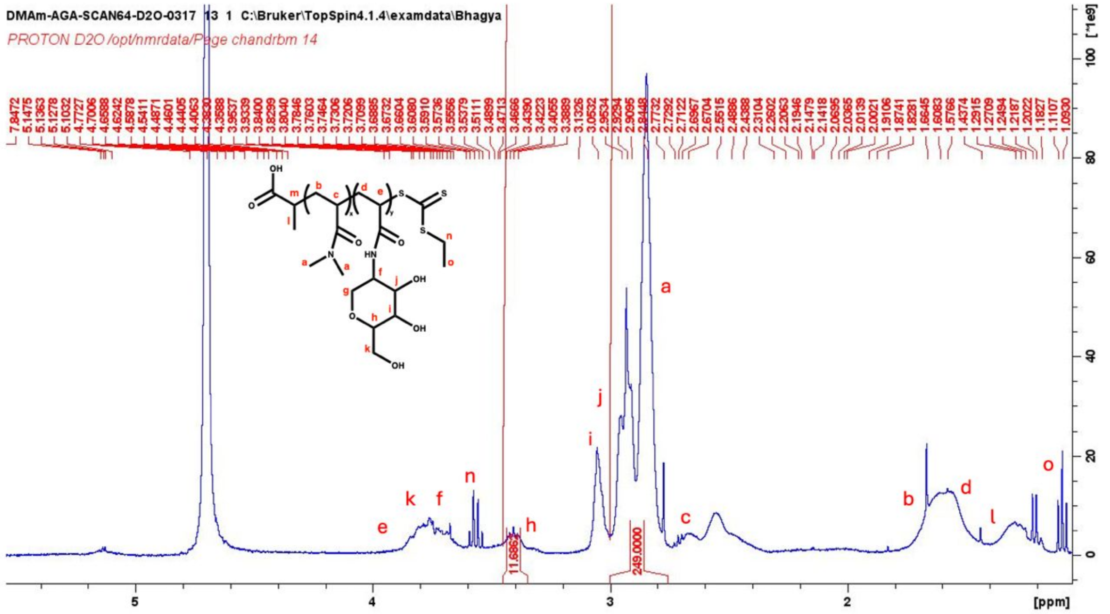

Figure S1. 1H NMR Spectra of (A)pDMAm DP40; (B)pDMAm DP20-b-NIPAm DP5; (C) pDMAm DP20-b-NIPAm DP10; (D) pDMAm DP40-b-NIPAM DP10; (E) pDMAPA DP20; (F) pDMAPA DP40; (G) pDMAPA DP20-b-NIPAm DP5; (H) pDMAPA DP20-b-NIPAm DP10; (I)

pDMAPA DP40-b-NIPAM DP10; (J) pDMAPA DP50; (K) pDMAPA DP40-b-AGA DP10; (L) pDMAm DP40-b-AGA DP10.

Table S2. Molecular weight Characteristics of polymers

**Sample**	**$M_{n\text{-}Th}$ (g/mol)**	**M_n (g/mol)[a]**	**PDI[a]**
pDMAm DP20	2193	1800	1.19
pDMAm DP40	4175	3400	1.12
pDMAm DP20-b-NIPAm DP5	2758	2000	1.19
pDMAm DP20-b-NIPAm DP10	3324	2600	1.26
pDMAm DP40-b-NIPAM DP10	5307	4700	1.27
pDMAPA DP20	3335	2900	1.34
pDMAPA DP40	6459	5000	1.37
pDMAPA DP20-b-NIPAm DP5	3900	4400	1.33
pDMAPA DP20-b-NIPAm DP10	4466	5100	1.33
pDMAPA DP40-b-NIPAM DP10	7591	8100	1.27
pDMAPA DP50	8021	7000	1.31
pDMAPA DP40-b-AGA DP10	8789	5300	1.33
pDMAm DP40-b-AGA DP10	6295	5000	1.15

[a] As determined by size exclusion chromatography.

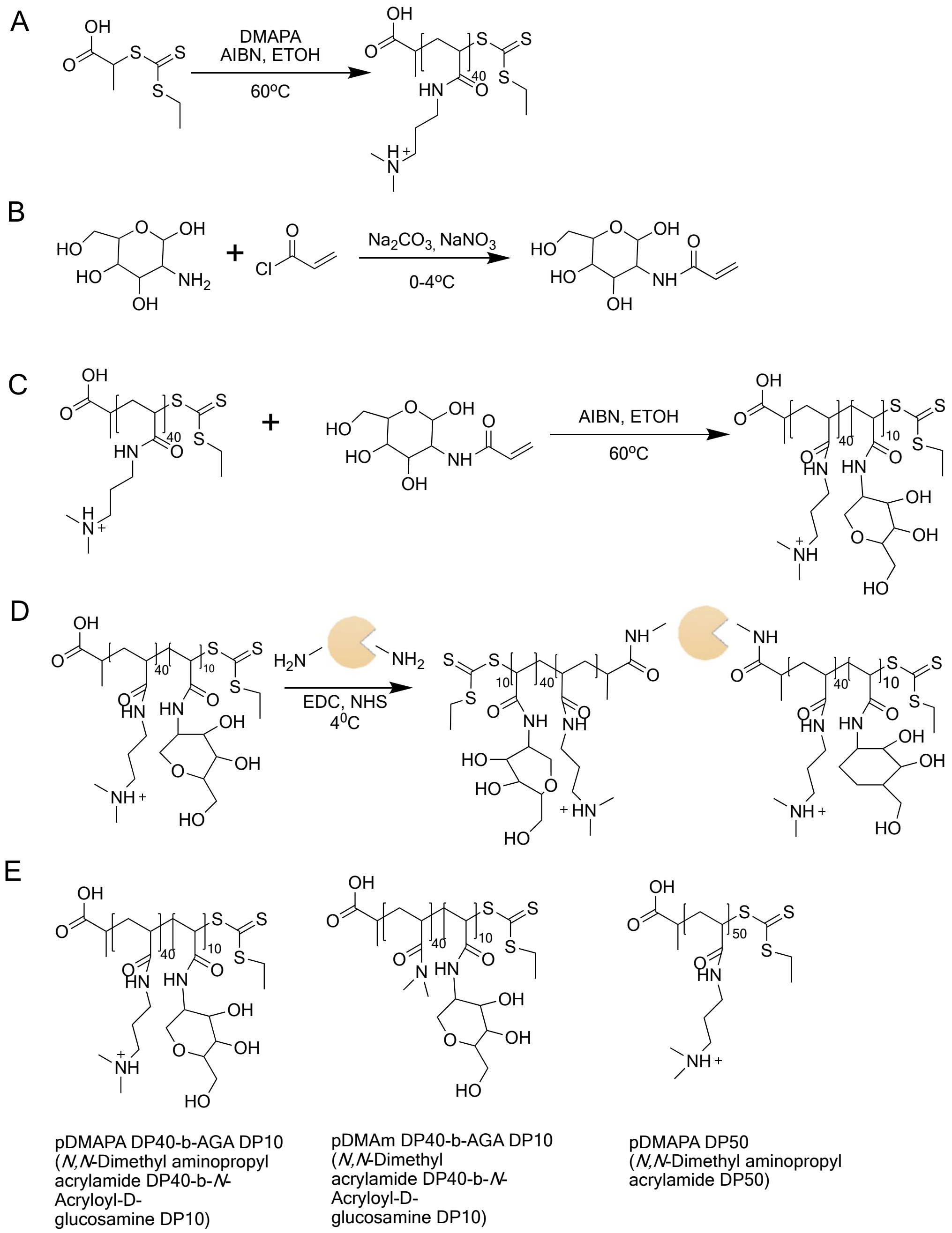

Scheme S1. (A) RAFT polymerization of pDMAPA DP40. (B) Synthesis of AGA. (C)"Grafting to" approach via EDC-NHS coupling of pDMAPA DP40-b-AGA DP10 to lysozyme protein. (D) Polymer used in lysozyme conjugation.

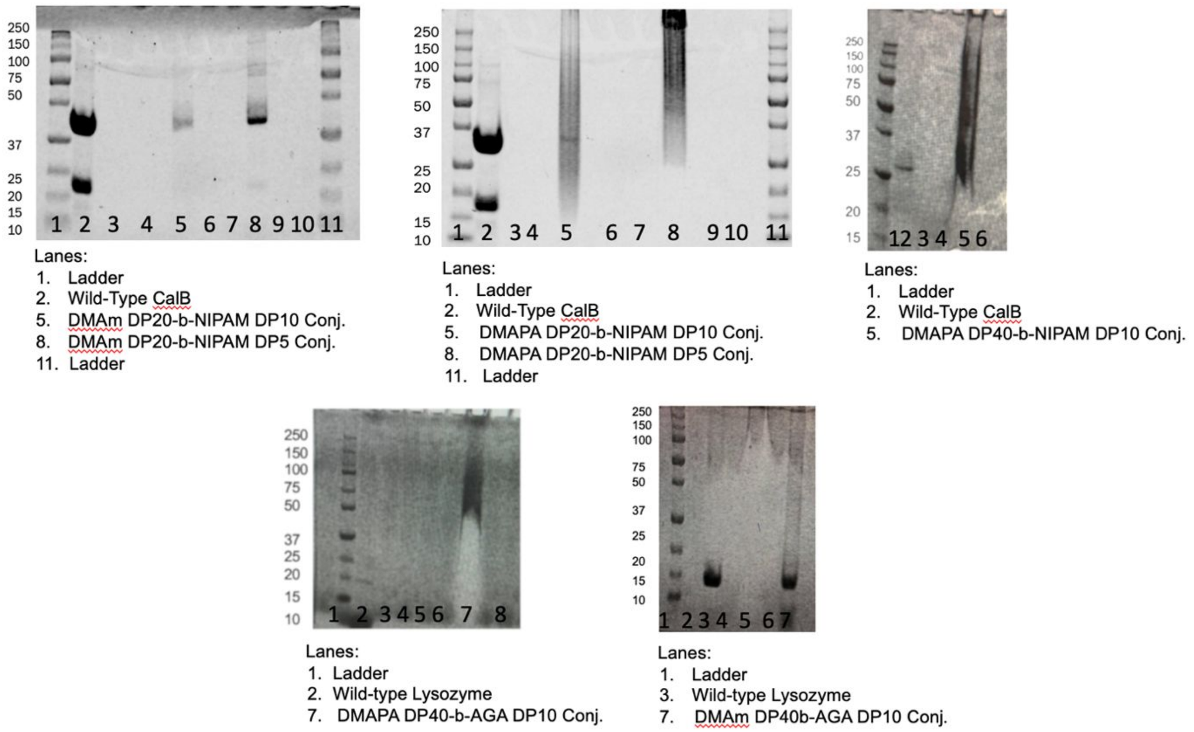

Figure S2. Characterization of conjugates using sodium dodecyl polyacrylamide gel electrophoresis (SDS-PAGE).

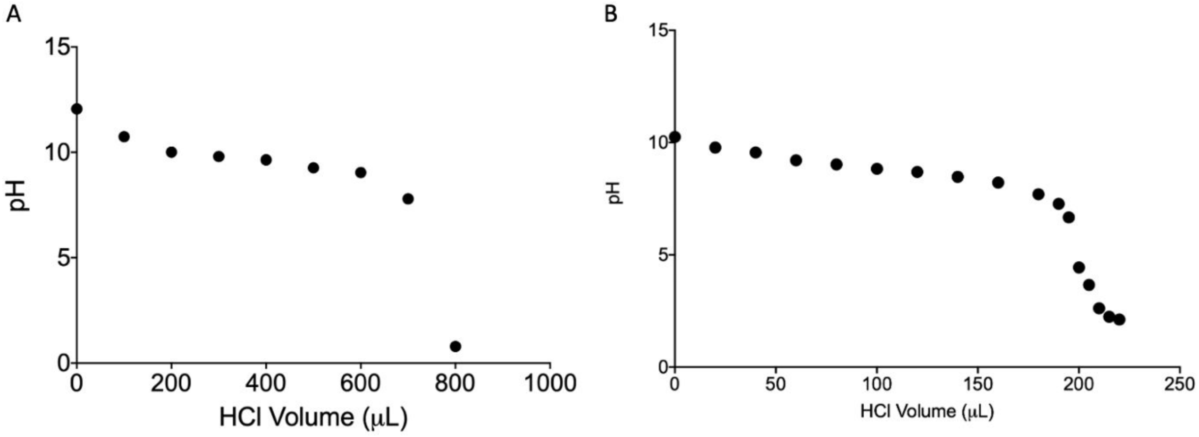

Figure S3. 6N HCl titration to 0.1 M DMAPA DP40-b-NIPAm DP10 (A) and 0.01 M DMAPA DP40-b-NIPAm DP10 (B) polymers.

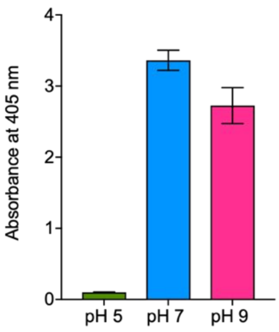

Figure S4. The activity of native CalB in three different pH systems (pH 5=green, pH 7=blue, and pH 9=pink).

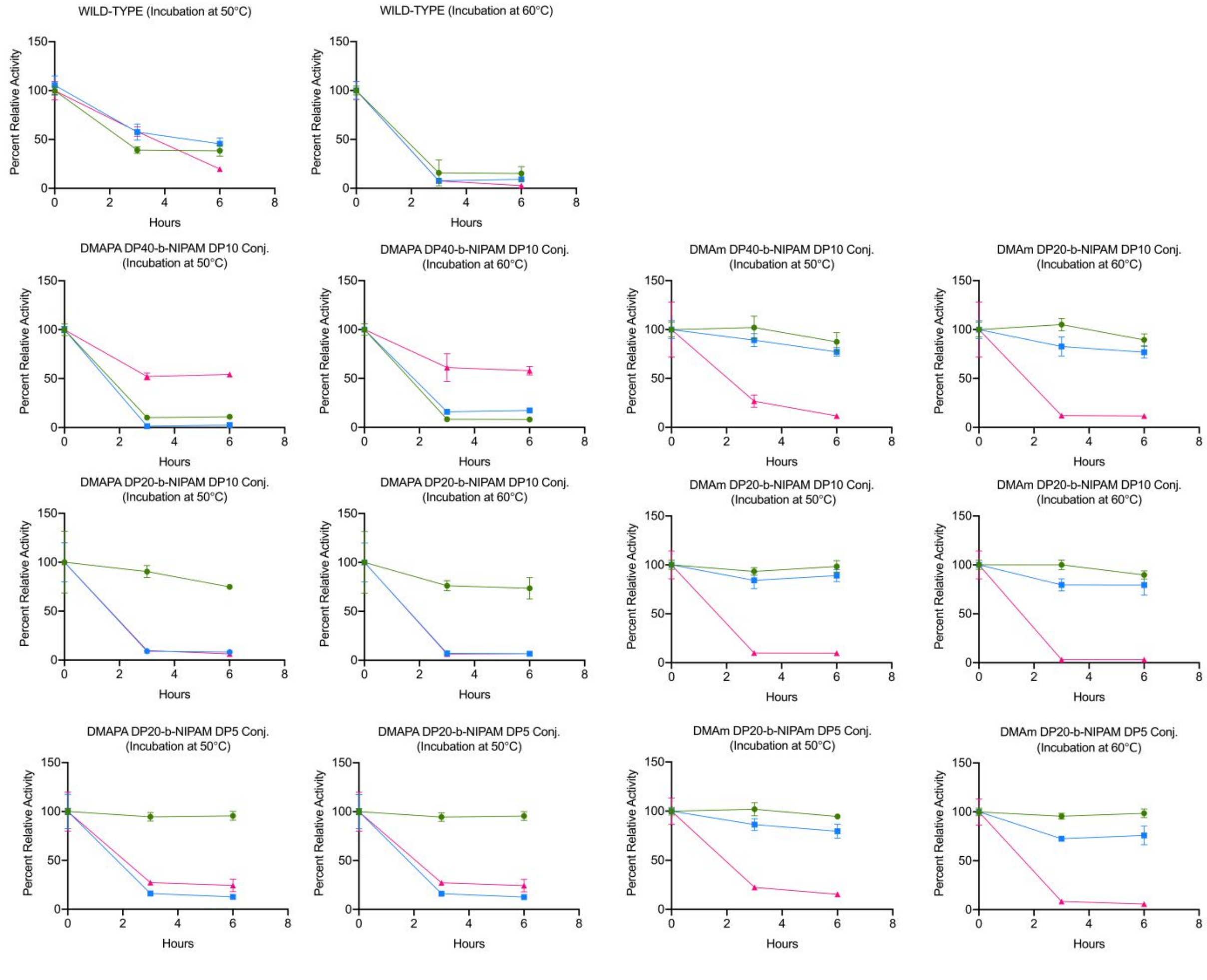

Figure S5. Functional thermal stability of wild-type lipase and its conjugate with different polymers. Each sample was incubated at 50℃ and 60℃ for two different time periods, 3 hours and

6 hours. The activity of each sample was normalized with respect to their initial activity at zero hours. Activities in pH 5, 7, and 9 are presented in green, blue, and pink, respectively. % Percent relative activity = (Absorbance of a sample at a particular hour/Absorbance of the sample at zero hours) x 100. Each data represents an average of triplicate ± Standard deviation.

A.

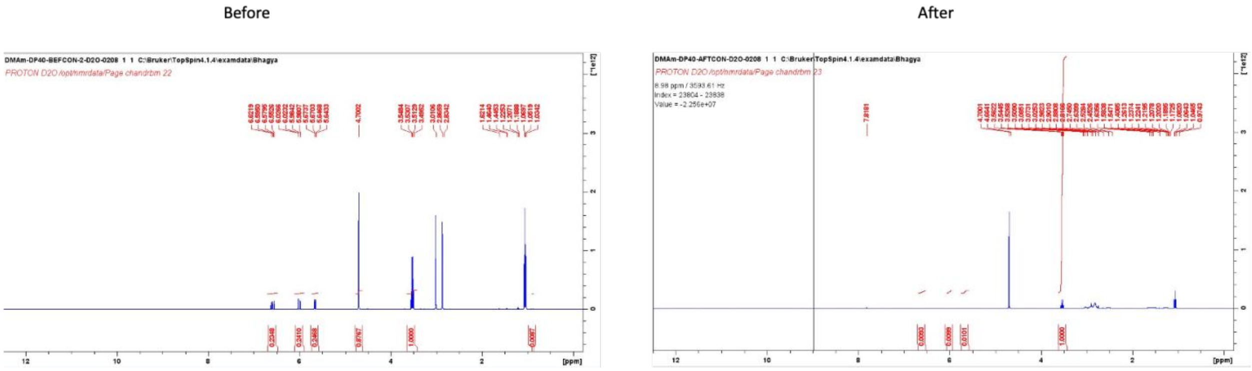

Figure S6. Conversion of pDMAm 40

B.

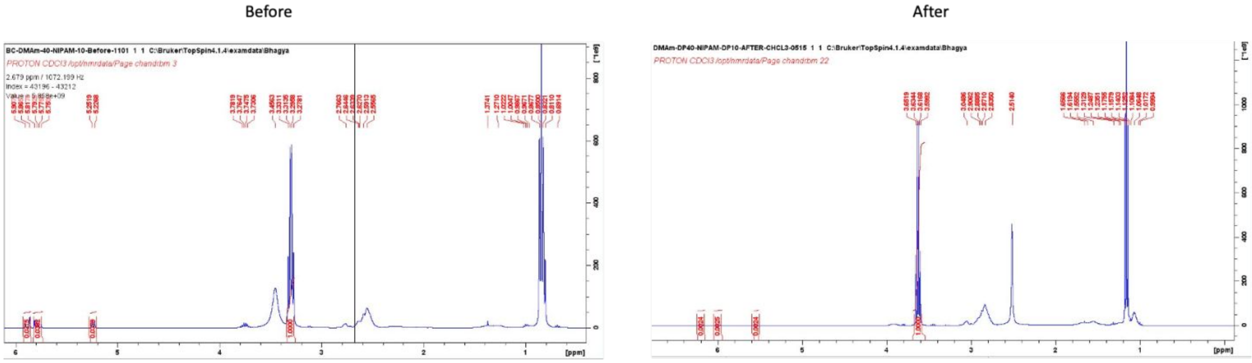

Figure S7. Conversion of pDMAm -DP40-NIPAm -DP10

C.

Before After

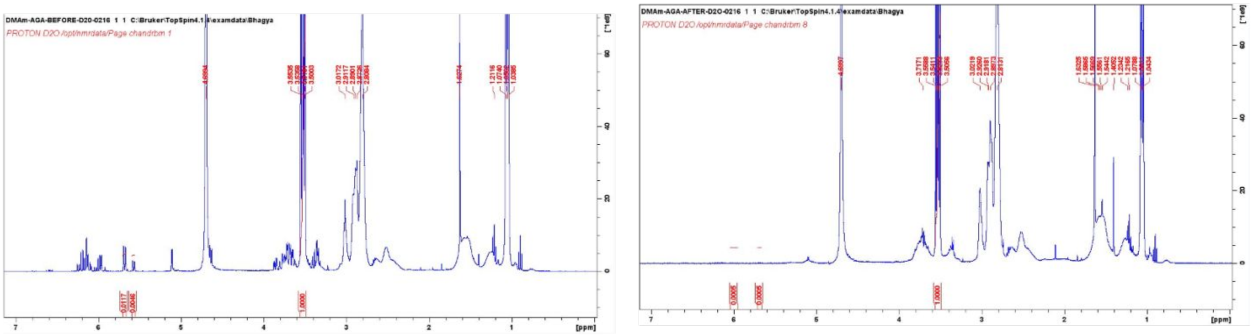

Figure S8. Conversion of pDMAm-DP40-AGA-DP10

D.

Before After

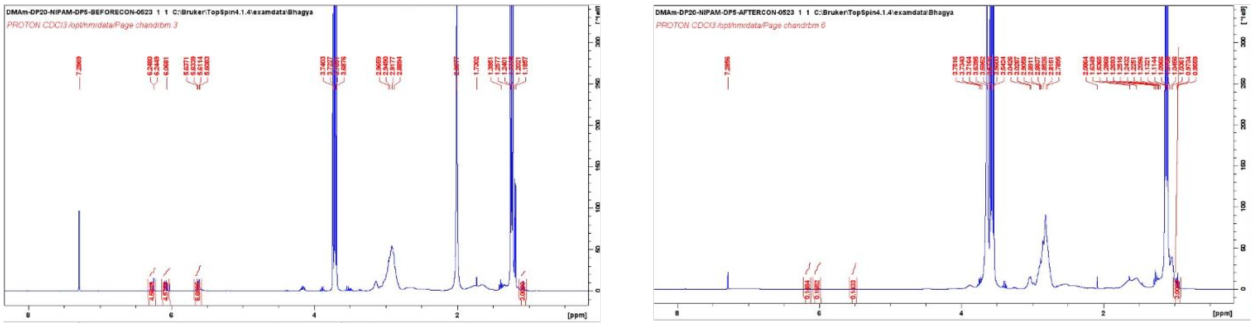

Figure S9. Conversion of pDMAm-DP20-NIPAm-DP5

E.

Before After

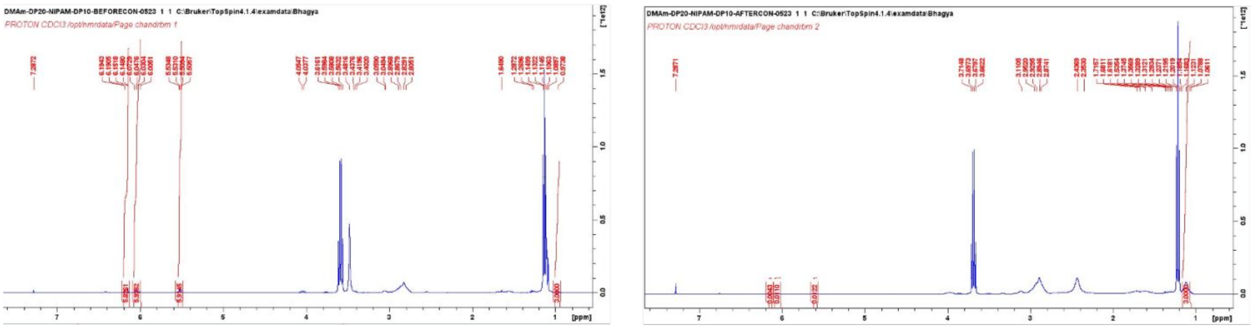

Figure S10. Conversion of pDMAm-DP20-NIPAm-DP10

F.

Before | After

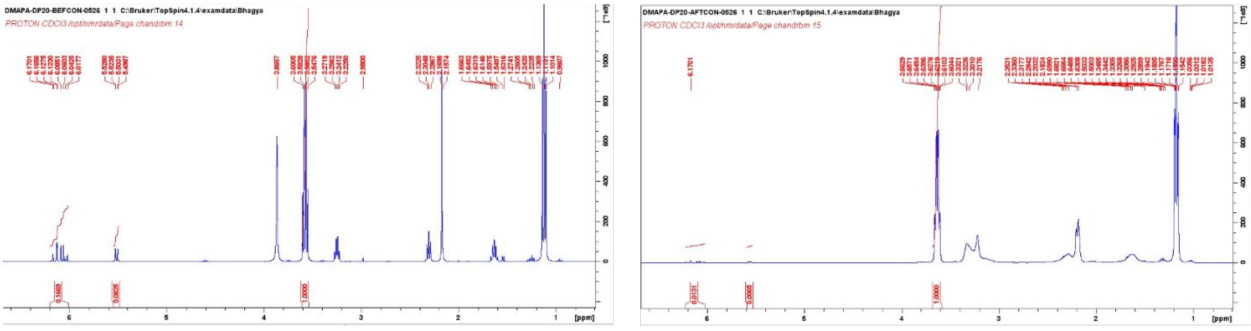

Figure S11. Conversion of pDMAPA-DP20

G.

Before | After

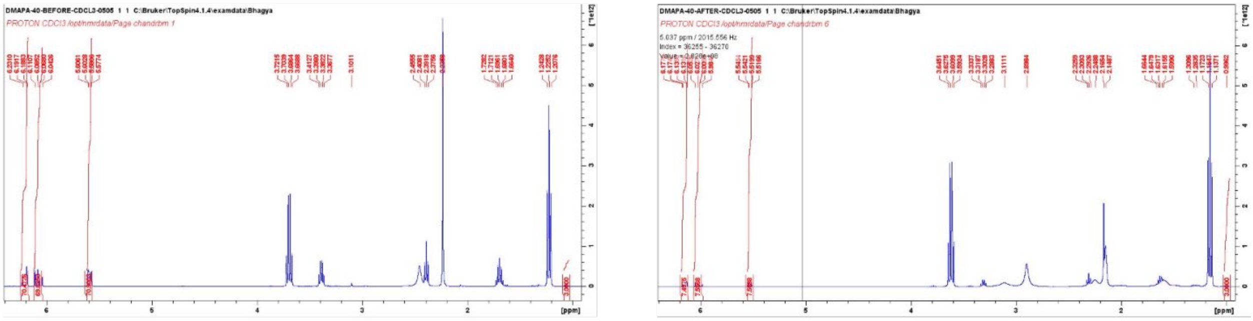

Figure S12. Conversion of pDMAPA-DP40

H.

Before | After

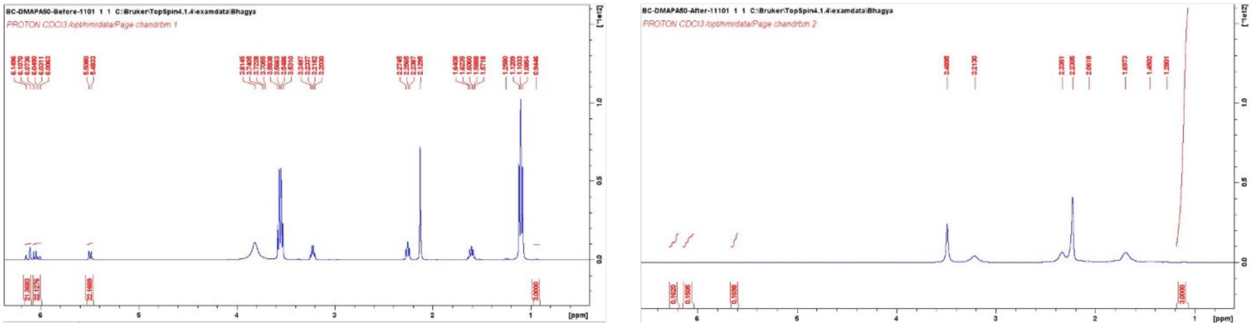

Figure S13. Conversion of pDMAPA-DP50

I.

Before | After

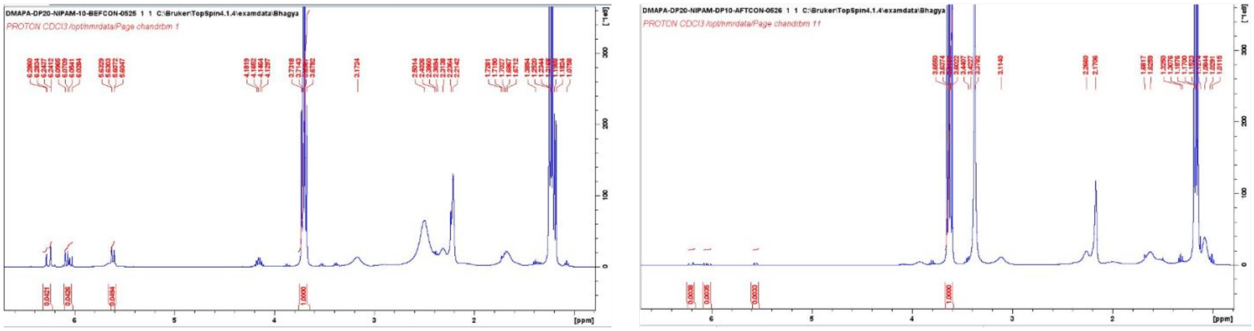

Figure S14. Conversion of pDMAPA-DP20-NIPAm-DP10

J.

Before | After

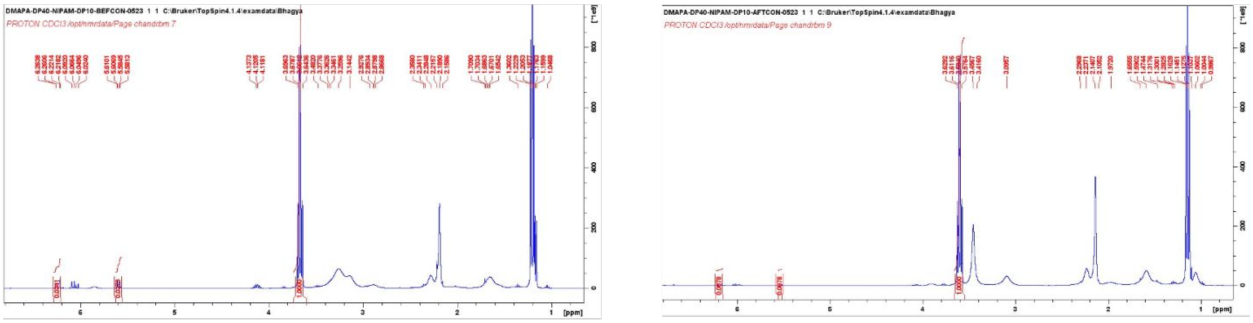

Figure S15. Conversion of pDMAPA-DP40-NIPAm-DP10

K.

Before | After

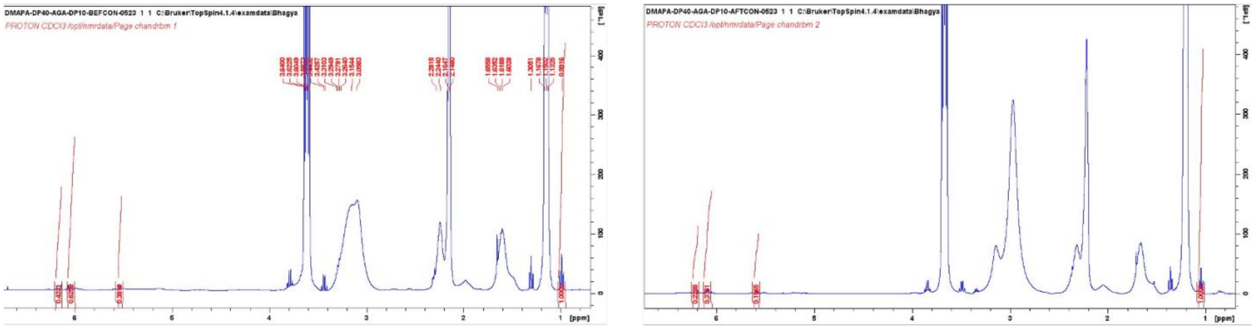

Figure S16. Conversion of pDMAPA-DP40-AGA-DP10

L.

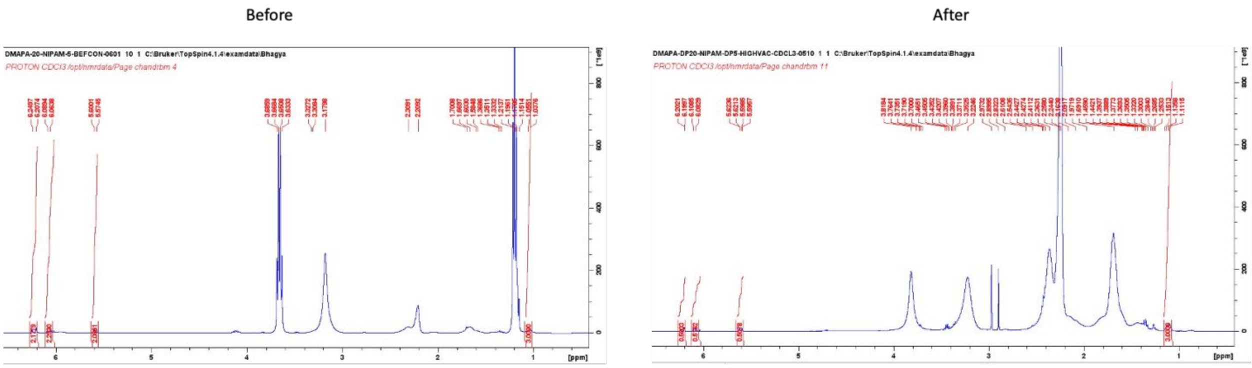

Figure S17. Conversion of pDMAPA-DP20-NIPAm-DP5

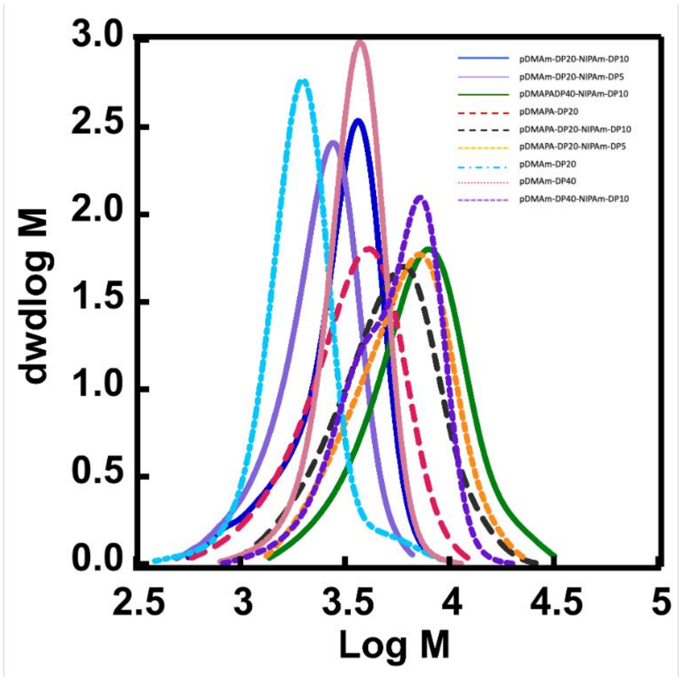

Figure S18. Polymer Characterization by Size Exclusion Chromatography

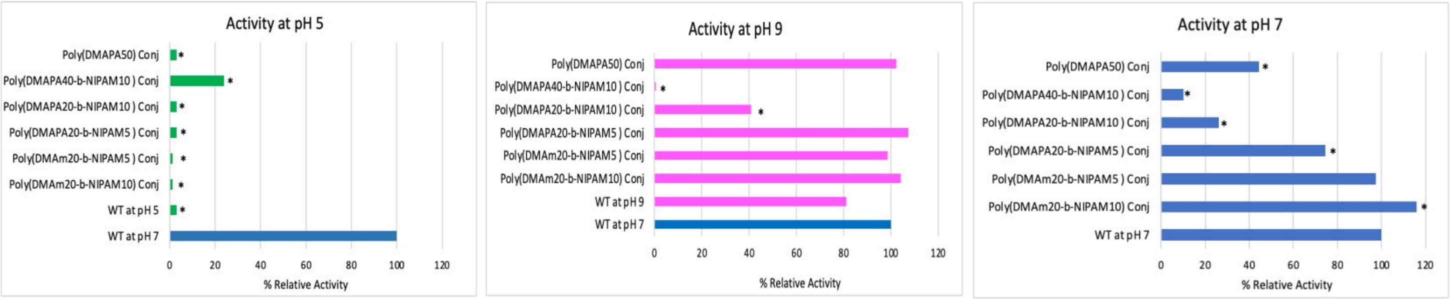

Figure S19 : Activity of each Cal B conjugate normalized against the WT Cal B activity at pH 7. Statical analysis by t – test: Paired two samples for mean , each WT and conjugate compared with WT protein at pH 7, * indicates $P < 0.05$

A. B. C.

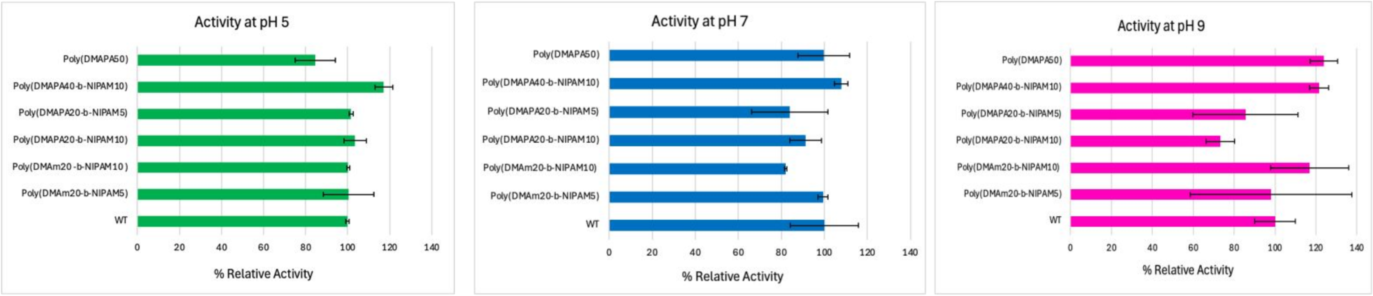

Figure S20 : Percent relative activity of control assay for wild type of lipase and its unconjugated polymer at different pH systems (A) Percent relative activity at pH 5 (Green); (B) Percent relative activity at pH 7 (Blue); (C) Percent relative activity at pH 9 (Pink). Activity was normalized with respect to wild type to lipase at related pH systems. %Percent relative activity = [Activity of sample at particular pH systems/Activity of Wild type of Lipase at that pH] x 100. Each data represents an average of duplicate ± Standard deviation.
